# High expression of RTEL1 predicates worse progression in gliomas and promotes tumorigenesis through JNK/ELK1 cascade

**DOI:** 10.1186/s12885-024-12134-8

**Published:** 2024-03-26

**Authors:** Guanjie Wang, Xiaojuan Ren, Jianying Li, Rongrong Cui, Xumin Zhao, Fang Sui, Juan Liu, Pu Chen, Qi Yang, Meiju Ji, Peng Hou, Ke Gao, Yiping Qu

**Affiliations:** 1 Key Laboratory for Tumor Precision Medicine of Shaanxi Province, Department of Endocrinology, The First Affiliated Hospital of Xi’an Jiaotong University, 710061 Xi’an, P.R. China; 2 Department of Oncology, Xi’an Central Hospital, 710061 Xi’an, P.R. China; 3 Department of Respiratory Disease, Xi’an Central Hospital, 710061 Xi’an, P.R. China; 4 Center for Translational Medicine, The First Affiliated Hospital of Xi’an Jiaotong University, 710061 Xi’an, P.R. China; 5 Department of Neurosurgery, The First Affiliated Hospital of Xi’an Jiaotong University, 710061 Xi’an, China; 6 Department of Radiation Oncology, The First Affiliated Hospital of Xi’an Jiaotong University, 710061 Xi’an, China

**Keywords:** RTEL1, Glioma, ROS, JNK signaling pathway, ELK1

## Abstract

Gliomas are the most common primary intracranial tumor worldwide. The maintenance of telomeres serves as an important biomarker of some subtypes of glioma. In order to investigate the biological role of RTEL1 in glioma. Relative telomere length (RTL) and RTEL1 mRNA was explored and regression analysis was performed to further examine the relationship of the RTL and the expression of RTEL1 with clinicopathological characteristics of glioma patients. We observed that high expression of RTEL1 is positively correlated with telomere length in glioma tissue, and serve as a poor prognostic factor in TERT wild-type patients. Further in vitro studies demonstrate that RTEL1 promoted proliferation, formation, migration and invasion ability of glioma cells. In addition, in vivo studies also revealed the oncogene role of RTEL1 in glioma. Further study using RNA sequence and phospho-specific antibody microarray assays identified JNK/ELK1 signaling was up-regulated by RTEL1 in glioma cells through ROS. In conclusion, our results suggested that RTEL1 promotes glioma tumorigenesis through JNK/ELK1 cascade and indicate that RTEL1 may be a prognostic biomarker in gliomas.

**Supplementary Information:**

The online version contains supplementary material available at 10.1186/s12885-024-12134-8.

## Introduction

Glioma is the most common tumor of central nervous system, and it persists approximately 35-43% of all intracranial tumors [1]. Although the prognosis for low grade gliomas has been improved, the outcomes for high grade gliomas are still grim, the median survival was less than 15 months [2]. Thus, there is a compelling need for developing additional therapeutic targets and treatment options. Telomere is a structure at the end of chromosome which plays a vital role to protect chromosome from degradation [3]. In humans, telomeres consist of 2–20 kb of double-stranded TTAGGG repeats with terminal 50- to 500-nucleotide long single-stranded G overhangs. Defects in the protection mechanisms of telomeres have been implicated in cancer [4]. Telomere length varies a lot among different tissues and cell lines. For instance, the telomere length was found to be longer at the blastocyst-stage when compared with early embryos [5]. In addition, telomere length was found significantly shortened in chronic myeloid leukemia [6]. However, in most solid tumors such as glioma [7], lung adenocarcinoma [8], neuroblastoma [9], bladder cancer [10], melanoma [11, 12], hepatocellular carcinoma [13], and kidney cancer [14], the telomere length was increased compared with normal tissues [15–18].

The maintenance of telomere length is affected by many factors, including the balance between competition factors. The dynamic balance factor of telomeres refers to the relative balance of telomere shortening and telomere elongation [19, 20]. The DNA helicase regulator of telomere length 1 (RTEL1) is an anti-recombinase that counteracts telomeric replication fork stalling by unwinding telomeric G quadruplex structures, thus preventing telomere fragility, and disassembling t-loops at certain stages of the cell cycle [21]. Previous study established that the shelterin protein TRF2 recruits RTEL1 to telomeres in S phase [22]. And RTEL1 can dismantles T Loops and counteracts telomeric G4-DNA to maintain telomere integrity, in the absence of RTEL1, T loops were inappropriately resolved by the SLX4 nuclease complex, resulting in loss of the telomere as a circle in RTEL1^−/−^ cells [23, 24]. Interestingly, some other researchers demonstrated that more than 70% transgenic mice that widely overexpress RTEL1 developed liver tumors that recapitulate many malignant features of human hepatocellular carcinoma (HCC) [25]. Previous studies have also linked RTEL1 mutations to several distinct types of human brain cancer and to Hoyeraal–Hreidarsson syndrome [26]. And a GWAS study of 1878 cases and 3670 healthy controls from four countries identified a significant association between the RTEL1 gene rs6010620 polymorphism and glioma risk [27]. Another study of 692 adult glioma cases and 3992 controls reported that two SNPs rs6010620 and rs4809324 of RTEL1 were significantly associated with the susceptibility to gliomas and astrocytomas [28]. However, the expression of RTEL1 in gliomas and its role in human cancers including glioma remains totally unclear. In this study, by a series of in vitro and in vivo assays, we are going to investigate the relationship between RTEL1 expression and clinicopathological characteristics in glioma patients, and to explore the mechanisms of RTEL1 caused in glioma tumorigenesis.

## Materials and methods

### Clinical samples

With approval of the institutional review board and human ethics committee of the First Affiliated Hospital of Xi’an Jiaotong University, 389 gliomas tissues from grade I to IV from 2010 to 2018 were enrolled in this study. And 50 meningoma samples from grade I were collected for non-glioma controls. Meningioma samples correspond to WHO I class however used as non-glioma control due to the lack of access to normal sample. Tumors were histopathologically classified according to the World Health Organization (WHO) classification based on 2007. WHO 2016 classification had not been used due to the lack of information regarding some molecular markers. All the patients involved in the current study have signed the informed consent forms.

### Cell culture and transfection

Human glioma cell lines used in the current study including A172, BT325, U87 and SF295 were purchased from the American Type Culture Collection (ATCC) (Manassas, VA, USA) and the Cell Bank of Animal Laboratory Center of Zhongshan University (Guangzhou, China), respectively. Cells were cultured at 37 °C in DMEM medium with 10% fetal bovine serum (FBS). The sequences of oligonucleotides of siRNAs targeting RTEL1 (si-RTEL1-1724 and si-RTEL1-3794) were shown in Supplementary Table S1. Transfection was performed when cells were at 50% confluence using Lipofectamine 3000 (Invitrogen, Grand Island, NY) according to the manufacturers’ instruction at a final concentration of 50 nM. Coding sequence (CDS) of RTEL1 was cloned into pcDNA3.1(−) mammalian expression vector to construct the RTEL ectopic expression plasmids. For RTEL1 overexpression, U87 and SF295 cells were transfected with the 2 μg RTEL1 plasmids or control plasmid at 70% confluence using X-treme GENE HP DNA Transfection Reagent (Invitrogen) according to the manufacturers’ instruction.

### Cell viability and colony formation assays

Cell viability was determined by the MTT assay. Briefly, cell (5000/well) were seeded and cultured in 96-well plates for 1 to 6 days at the indicated times, 20 μl of 0.5 mg/ml MTT (Sigma, Saint Louis, MO) was added into the medium and incubated for 4 h, followed by adding 150 μl of DMSO for additional 15 min. The plates were then read on a microplate reader using a test wavelength of 570 nm and a reference wavelength of 670 nm. Three triplicates were done to determine each data point. A bottom layer of 2 ml DMEM supplemented with 07% agar and 10% FBS and a top layer of 1 ml DMEM supplemented with 035% agar and 10% FBS were added in 6-well plates, which contained 2,000 cells/well and were then incubated for 2–3 weeks at 37 °C Subsequently, with a diameter ≥ 200 μm, the total number and sizes of colonies were calculated using a light microscope (Olympus Corporation) in > 5 fields per well for a total of 15 fields in triplicate experiments.

### Cell migration and invasion assays

Cell migration and invasion assays were assessed by transwell chambers (8.0 μm pore size; Millipore, MA) pre-coated with rat tail tendon collagen type 1 (0.5 mg/mL) on the lower surface. For cell invasion assay, chambers were coated with Matrigel (4 × dilution; 15 μl/well; BD Bioscience, NJ). Cell clones stably transfected with pcDNA3.1-POSTN or empty vector were starved overnight and then seeded in the upper chamber at a density of 1 × 10^6^ cells/ml in 200 μl of medium containing 0.5% FBS. Medium with 10% FBS (1 ml) was added to the lower chamber. After a 24- or 48-h incubation (depending on the cell type), non-migrating or non-invading cells in the upper chamber were removed using a cotton swab, and migrating or invading cells were then fixed in 100% methanol and stained with crystal violet solution (0.5% crystal violet in 2% ethanol). Photographs were taken randomly for 5 fields of each membrane. The number of migrating/invading cells was expressed as the average number of cells per microscopic field over 5 fields.

### Cell cycle and apoptosis assays

For cell cycle assay, cells transiently transfected with different plasmids were harvested at 48 or 72 h when the confluence reached ∼90%, and washed twice with PBS. Cells were then fixed in ice-cold 70% ethanol for at least 30 min, and stained with propidium iodide solution (50 μg/mL propidium iodide, 50 μg/mL RNase A, 0.1% Triton-X, 0.1 mM EDTA). Cell cycle distributions were assessed based on DNA contents by FACS using a Flow Cytometer (BD Biosciences, NJ). For apoptosis analysis, the indicated cells were harvested, washed with PBS, suspended in binding buffer, and sequentially stained with Annexin V-FITC Detection Kit (Roche Applied Science, Penzberg, Germany) by flow cytometer according to the manufacturer’s protocol. Each experiment was performed in triplicate.

### RNA sequence and microarray

To determine the effect of RTEL1 expression on the transcriptome of U87 and SF295, RNA-sequence assay and hospho-specific antibody microarray was performed by Genenergy Bio-technology (Shanghai, Inc. https://www.genenergy.cn). Heatmaps are commonly used to visualize RNA-seq results. Heatmaps showing the differentially expressed genes were used to visualize RNA-seq results and the common 1131 genes of these two cell lines was selected (Supplementary Table S2). Furthermore, to assess whether RTEL1 alters the downstream signaling events of phosphorylation, a phospho-specific antibody microarray targeting 269 proteins of classical tumor pathways was performed in both U87 and SF295 cells by Wayen Biotechnologies (Shanghai, Inc. https://www.wayenbio.com/) (Supplementary Table S3).

### Real-time polymerase chain reaction (qPCR)

Total RNA from tissues and cell lines were extracted using Trizol reagent (Takara Inc., Dalian, P.R. China) following the manufacturer’s protocol. The cDNA was synthesized with 500 ng total RNA by using PrimeScript RT reagent Kit (Takara Inc., Dalian, P.R. China). Quantitative RT-PCR (qRT-PCR) was carried out on a CFX96 Thermal Cycler DiceTM real-time PCR system (Bio-Rad Laboratories, Inc., CA) using SYBR Premix Ex TaqTM (Takara Inc., Dalian, P.R. China). The mRNA expression of the indicated genes was normalized to 18 S rRNA cDNA. Each sample was run in triplicate. Primer pairs used in this study were presented in Supplementary Table S4. In addition, the relative telomere length (RTL) of each sample was determined by qPCR as previously described.

### Western blotting

Cells were lysed in prechilled RIPA buffer (Cell Signaling Technology, Inc) containing protease inhibitors The protein concentration was determined using A280 absorbance measurements by NanoDrop2000 Ultra Micro Spectrophotometer (Thermo Fisher Scientific, Inc) Equal amounts (100 μg per lane) of protein lysates were separated by 10% SDS-PAGE and transferred to PVDF membranes (Roche Diagnostics GmbH), the membranes of each blots were cut prior to hybridisation with antibodies, and then blocked with 10% skimmed milk at 37 °C for 2 h Next, the membranes were incubated with the indicated primary antibodies at 4 °C overnight After incubation of the membranes with species-specific HRP-conjugated secondary antibodies, goat anti-rabbit antibody (1:3,000; cat no TA130023; OriGene Technologies, Inc) or goat anti-mouse antibody (1:3,000; TA130004; OriGene Technologies, Inc), for 2 h at 37 °C, the Western Bright ECL detection system (Advansta, Inc) was used to visualize the immunoblotting signals. Primary antibodies were all purchased from Abcam, Santa cruz and CST and mainly contain RTEL1 (1:500; ab85557), phospho-ELK1 (1:1000; sc-8406), total-ELK1 (1:1000; sc-365,876), phospho-JNK (1:1000; #4668), total-JNK (1:1000; #9252), phospho-c-JUN (1:1000; #9165), total-c-JUN (1:1000; #3270) and GAPDH (1:2000; #2118).

### Reactive oxygen species (ROS) detection

The fluorescent probe Dichloro-dihydro-fluorescein diacetate (DCFH-DA) (Invitrogen, CA, USA) was used to detect the ROS. Both U87 and SF295 cells were treated with siRNAs targeting RTEL1, NAC (N-acetyl-L-cysteine) or combination of the both. Then the cells with indicated treatments were collected and suspended in serum-free medium. DCFH-DA were then added to each sample at a final concentration of 10 μM and incubated at 37 °C for 30 min. Subsequently, fluorescence was detected by flow cytometer.

### Immunohistochemistry (IHC)

Paraffin-embedded sections (5 μm) were deparaffinized and rehydrated in a graded series of ethanol, and washed in distilled water. After antigen retrieval and blocking, the sections were incubated with RTEL1 (1:100; ab85557), p-JNK (1:200; #4668), p-c-JUN (1:200; #9165), p-ELK1 (1:200; sc-8406) and Ki67 (1:200; sc-23,900) antibodies overnight at 4 °C. Immunodetection was performed with the Streptavidin-Peroxidase system (ZSGB-bio, Beijing China) according the manufacture’s protocol. 3,3′-diaminobenzidine was used as chromogen. Slides were then counterstained with hematoxylin. To ensure the comparability of immunohistochemical staining, a common reference standard was included to serve as an internal or intra-assay control in each batch.

### Animal studies

Twelve male athymic nude mice (4-week-old) were purchased from Xi’an Jiaotong university animal center and randomly divided into two groups (six mice per group). Then 1 × 10^7^ SF295 cells stably knocking down RTEL1 or control cells were injected into the right armpit region of the nude mice to establish tumor xenografts. Tumor volume was measured every 2 days and calculated by the formula (length × width^2^ × 0.5). Ten days after implantation, mice were sacrificed and xenograft tumors were harvested and weighted. All animals’ experimental procedures were approved by the Laboratory Animal Center of Xi’an Jiaotong University.

### Statistical analysis

Data are presented as mean ± standard deviation (SD) and statistics analyses were performed by the SPSS 19.0 (Chicago, IL, USA) software. One-way ANOVA or two-tailed Student’s t-test was used for comparisons between groups. Univariate and multivariate Cox regression analyses were performed to evaluate the prognostic value of RTEL1. Survival curves were constructed using the Kaplan–Meier method and statistical analysis was performed via the Log-rank test. A *p* value of < 0.05 was considered statistically significant.

## Results

### Telomere length positively correlated with RTEL1 expression predicated worse progression in gliomas

**Fig. 1:**
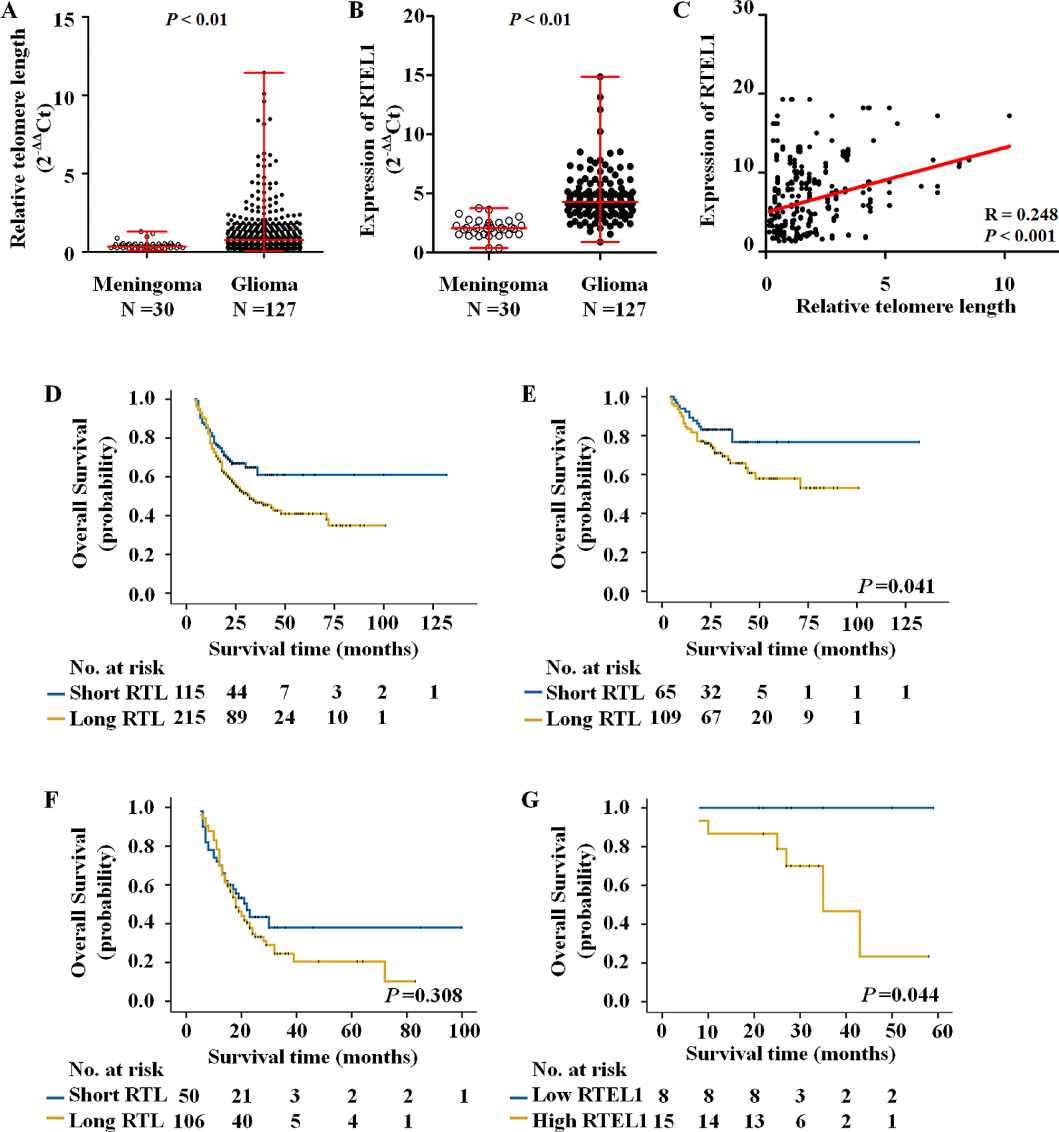
Telomere length correlated with the expression of RTEL1 predicated worse progression of glioma patients. The telomere length (**A**) and mRNA expression (**B**) was analyzed by qRT-PCR in gliomas with 18 S rRNA as the normalized controls (T; *n* = 127) and non-glioma control tissues (NT; *n* = 50). 2^−ΔΔCt^ and two-tailed t-test was used to analyze the Statistical differences. (**C**) The association of telomere length with expression of RTEL1 in glioma tissues was assessed by linear regression analysis. *R* = 0.248; *p* < 0.001. By using Kaplan–Meier and Univariate Cox regression, long RTL caused a poorer overall survival than short RTL in all patients (*n* = 330) (**D**) and in the patients with grade I-II gliomas (*n* = 174) (**E**) but not in the patients with grade III-IV gliomas (*n* = 156) (**F**). (**G**) High expression of RTEL1 caused a poorer overall survival in TERT wild-type patients (*n* = 238). Data were expressed as mean ± SD. ** *p* < 0.01

In order to exlpore the role of RTEL1 in gliomas, we first tested relative telomere length (RTL) and RTEL1 mRNA in gliomas and non-glioma control samples by using quantitative real-time RT-PCR (qRT-PCR). As shown in Fig. 1A and B, both telomere length and RTEL1 mRNA in gliomas is significantly elevated compared to those in the meningoma control tissues. Linear regression analysis was carried out and we found positive correlation between telomere length and the mRNA level of RTEL1 (*R* = 0.248, *p* < 0.001; Fig. 1C).

To further examine the relationship of the RTL and the expression of RTEL1 with clinicopathological characteristics of glioma patients, we calculated 95% CI (0.15–0.62) of RTL, (0.87–3.98) of RTEL1 mRNA in non-glioma control tissue, then define 0.62 and 3.98 as cut-off values of RTL and RTEL1 mRNA, respectively. As shown in Supplementary Table S5, by using univariate regression analysis, we found that telomere length is relevant with recurrence, and higher level of RTEL1 mRNA is more prevalent in male patients (*p* < 0.01), with favorite localization (*p* < 0.05) and pathological diagnosis, most significantly, repelling with TERT promoter mutations.

In order to assess the independent association of the RTL and RTEL1expression with age, gender, WHO grade, pathological diagnosis, recurrence, seizures and KPS, we conducted a multivariable logistic regression. As shown in Supplementary Table S6, long RTL remained closely associated with recurrence (OR = 1.70, 95% CI = 1.00-2.89, *p* < 0.05), and higher level of RTEL1 mRNA also associated with gender (OR = 2.23, 95% CI = 1.77–3.24, *p* = 0.012) and pathological diagnosis (OR = 3.19, 95% CI = 1.15–5.22, *p* = 0.002) in glioma patients.

Kaplan-Meier survival analysis was then used to further validate the effect of RTL and RTEL1expression on survival. The data showed that there was a significantly more favorite survival in the patients with short RTL than those with long RTL in all 330 glioma patients (*p* = 0.0027) (Fig. 1D). Further stratified analysis was conducted, and we found a similar result in 174 low grade gliomas (WHO 1 and 2, *p* < 0.05; Fig. 1E), while no significant difference was found in 156 high grade gliomas (*p* > 0.05; Fig. 1F). However, when we analysis the effect of level of RTEL1 mRNA on survival, there was no significant difference between high and low level of RTEL1 mRNA groups (data not shown). One possible explanation is, RTEL1 expression may finally differ RTL, on the other hand, there are more than one factors that influence the RTL, such as TERT promoter mutations. When we exclude patients carried TERT promoter mutations, a significant difference was found between high and low level of RTEL1 mRNA groups in 238 glioma patients (*p* < 0.05; Fig. 1G).

### RTEL1 knockdown inhibits the malignant biological properties of glioma cells

**Fig. 2:**
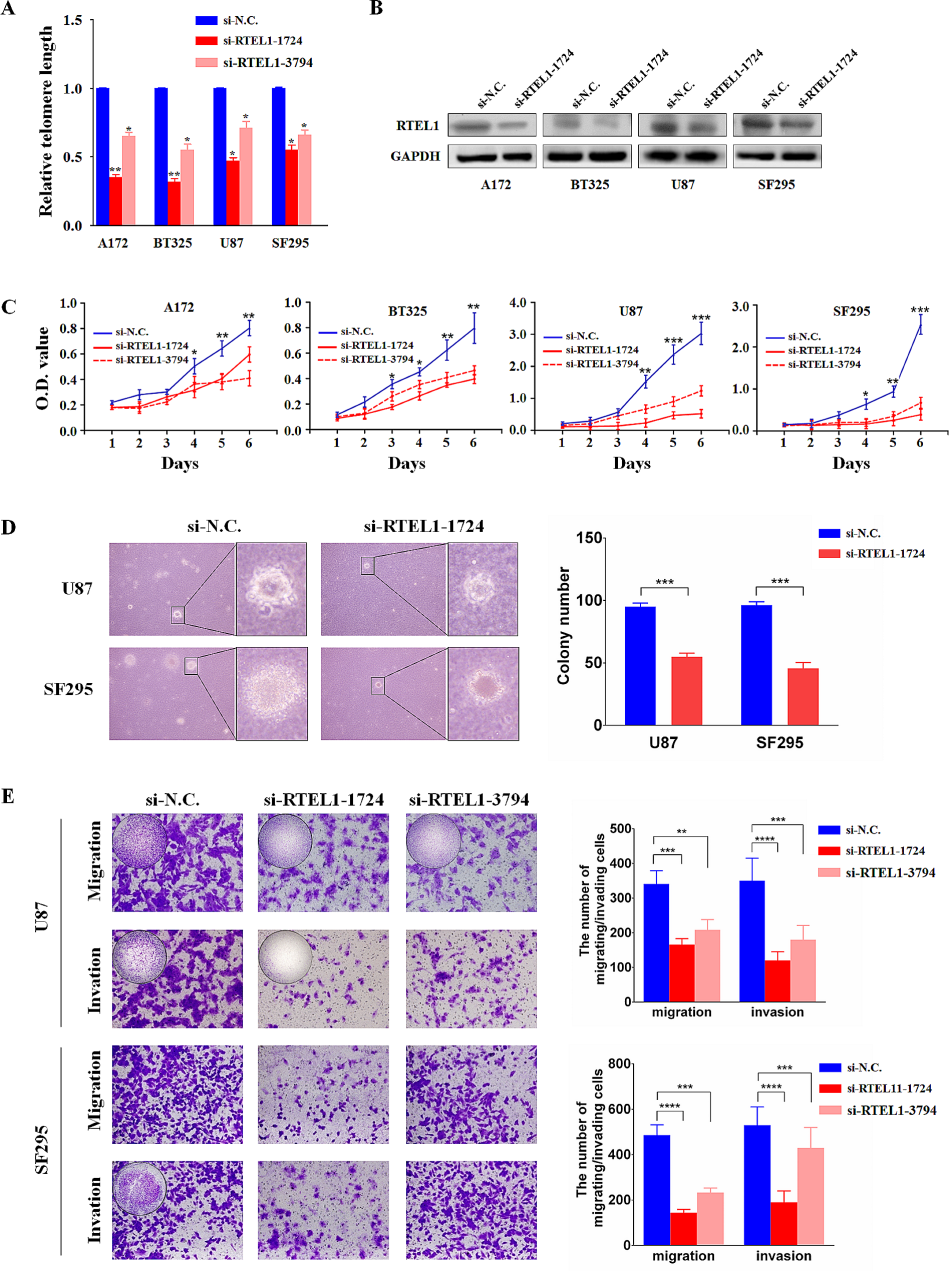
RTEL1 knockdown inhibits the malignant biological properties of glioma. Inhibition efficiency of the two different specific short interfering RNAs (siRNAs) targeting RTEL1 (si-RTEL1-1724 and − 3794) was determined by quantitative RT-PCR (qRT-PCR) (**A**) and Western blot assays (**B**) with 18 S rRNA and GAPDH as the normalized controls. (**C**) MTT assay was used to examine the effect of RTEL1 knockdown on glioma cell proliferation. RTEL1 knockdown significantly inhibited cell proliferation compared to the negative control. (**D**) The impact of RTEL1 knockdown on colony formation ability of glioma cells using soft agar. And RTEL1 knockdown significantly inhibited colony formation ability. (**E**) Cell migration and invasion assays were assessed by transwell chambers. And RTEL1 knockdown decreased migration and invasion potential of glioma cells. Mann–Whitney U test was used and data were expressed as mean ± SD. ** *p* < 0.01, *** *p* < 0.001

To explore the role of RTEL1 in glioma tumorigenesis, a series of in vitro studies were performed with gain-of-function and loss-of-function of RTEL1 in glioma cells including A172, BT325, U87 and SF295. First, we determined the inhibition efficiency of the two different specific short interfering RNAs (siRNAs) targeting RTEL1 (si-RTEL1-1724 and − 3794) by quantitative RT-PCR (qRT-PCR) and western blot assays (Fig. 2A and B). We found that RTEL1 knockdown significantly inhibited cell proliferation and colony formation of glioma cells compared to the negative control (Fig. 2C and D), which demonstrated the carcinogenic role of RTEL1 in glioma cells. Then, we analyzed the ability of migration and invasion of glioma cells by transwell assays. As shown in Fig. 2E, RTEL1 knockdown significantly decreased the number of migrating and invading cells compared to si-N.C. These findings proved that RTEL1 was closely associated with metastatic phenotypes of glioma cells.

### RTEL1 knockdown promotes cell cycle arrest and apoptosis of glioma cells

**Fig. 3:**
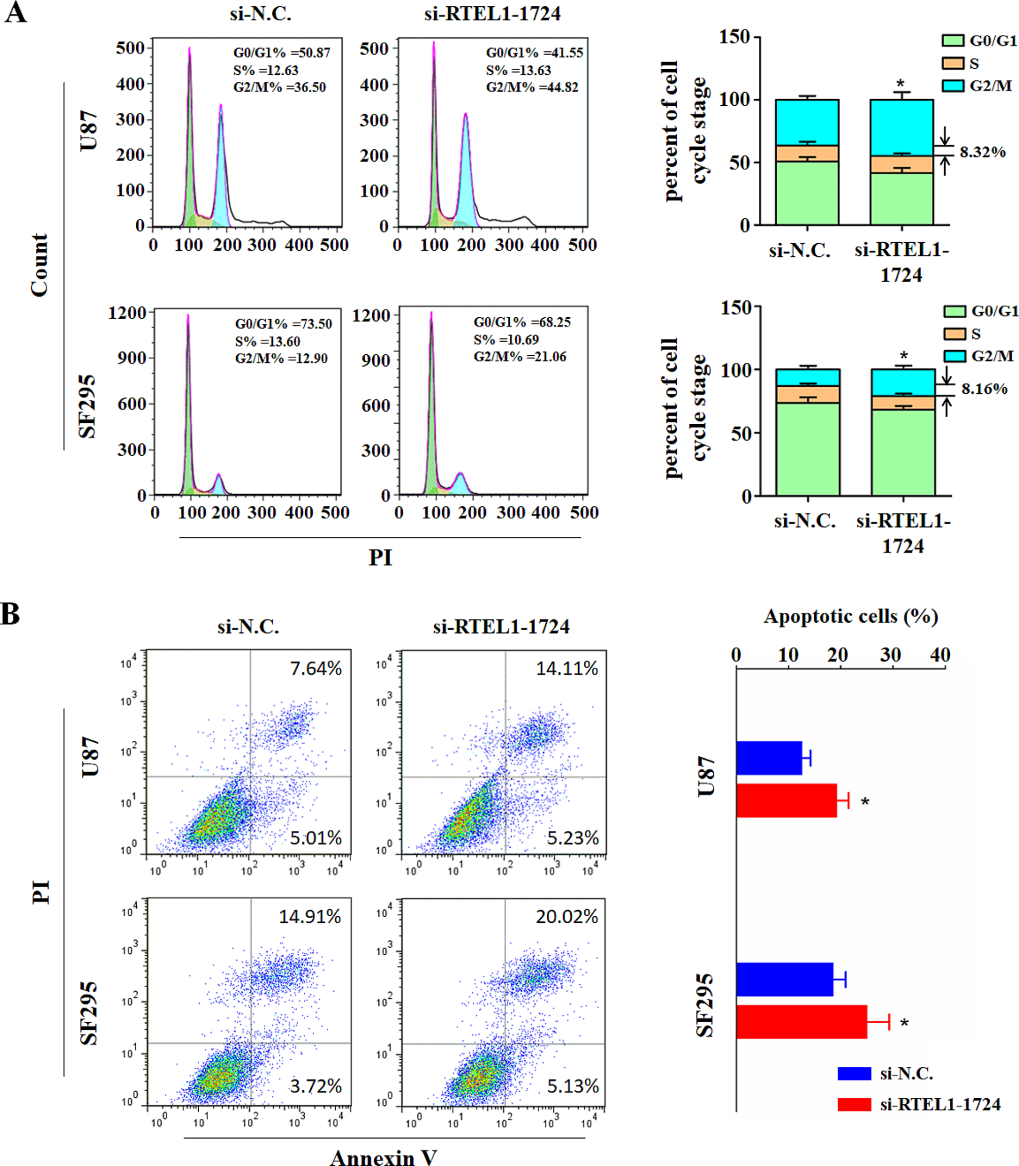
RTEL1 knockdown promotes cell cycle arrest and apoptosis of glioma. (**A**) Flow cytometry was used to analyze the effect of transient transfection of indicated siRNAs on cell cycle distributions. RTEL1 knockdown caused G2/M phase arrested in U87 and SF295 cells. (**B**) The apoptosis of the indicated cells transiently transfected with siRNAs was measured by flow cytometry using the Annexin V-FITC/PI Apoptosis Detection Kit. RTEL1 knockdown promoted cell apoptosis in U87 and SF295 cells. si-N.C represents siRNA of non-target as controls; si-RTEL1-1724 represents siRNA of RTEL1; Mann–Whitney U test was used and data were expressed as mean ± SD. * *p* < 0.01

In addition, we also evaluated the effect of RTEL1 knockdown on glioma cell cycle distributions and apoptosis. Compared to the si-N.C cells, cell cycle was arrested at the G2/M phase in the RTEL1 siRNAs-transfected cells. In detail, the percentage of G2/M phase in U87 and SF295 cells was increased from 36.5 ± 4.6–44.82% ± 9.4% and 12.90 ± 3.0% to 21.06 ± 4.5% (*p* < 0.05), respectively (Fig. 3A). Furthermore, RTEL1 knockdown dramatically promoted glioma cell apoptosis relative to the si-N.C. As shown in Fig. 3B. The proportion of apoptotic cells was increased from 12.65 ± 2.81% to 19.43 ± 3.48% in U87 cells (*p* < 0.05), and from 18.73 ± 3.41% to 25.15 ± 5.86% in SF295 cells (*p* < 0.05), respectively.

### ELK1 is a key downstream target gene regulated by RTEL1 in glioma cells

**Fig. 4:**
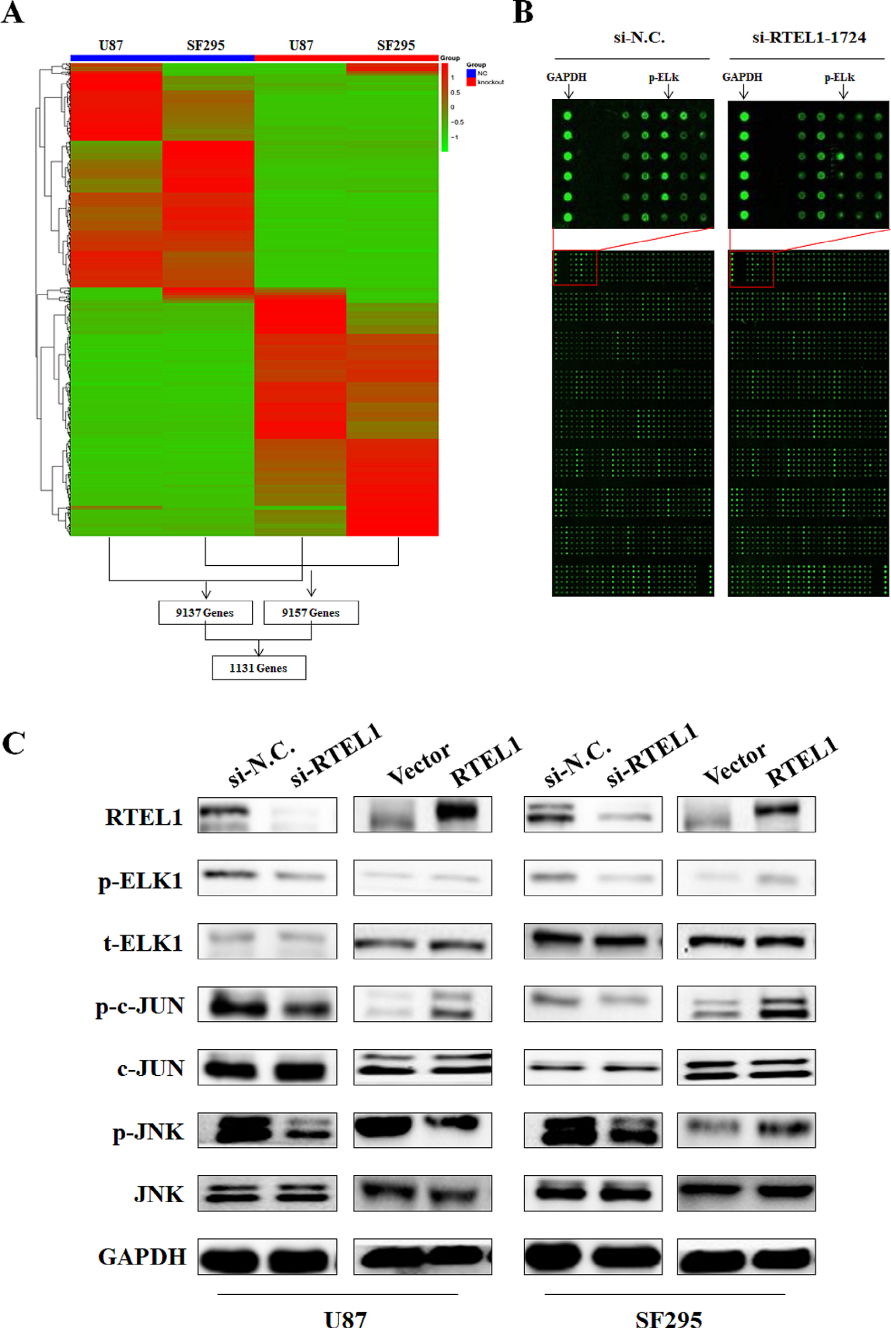
Identification downstream target genes regulated by RTEL1. (**A**) Heatmap plot of differentially expressed genes in RTEL1-depleted U87 and SF297 cells and the common 1131 genes of these two cell lines was selected performed by Genenergy Bio-technology (Shanghai, Inc. https://www.genenergy.cn). (**B**) Phospho-specific antibody microarray was used and Fluorescence-based assay for screening of differentially expressed proteins in RTEL1-depleted U87 cells performed by Wayen Biotechnologies (Shanghai, Inc. https://www.wayenbio.com/). (C) Western blot analysis of phosphorylated ELK1 and the JNK signaling pathway in RTEL1-depleted U87 and SF297 cells with GAPDH as the normalized controls. si-N.C represents siRNA of non-target as controls; si-RTEL1-1724 represents siRNA of RTEL1.

To determine the effect of RTEL1 expression on the transcriptome of U87, SF295, RNA-sequence assay was performed. Overall, RTEL1 knockdown resulted in upregulation or downregulation of numerous target genes. We identified 9137 and 9157 differentially expressed genes in si-RTEL1 transfected U87, and SF295 cells compared to control cells, respectively. By comparing differentially expressed genes in the two glioma cell lines, we identified 1131 genes which are regulated by RTEL1 in both cell lines (Fig. 4A and Supplementary Table S2). To further assess whether RTEL1 alters the downstream signaling events of phosphorylation, a phospho-specific antibody microarray targeting 269 proteins of classical tumor pathways was performed. This antibody assay included 131 pairs of antibodies which can identify phosphorylated target site and un-phosphorylated target site equally. Using a fold change higher than 2, we identified 2 pairs of phosphorylation sites of tyrosine, namely ELK1 (Ser383) and p53 (Ser315), were regulated by RTEL1 in U87 cells (Fig. 4B and Supplementary Table S3). It is well documented that ELK1 are known phosphorylation substrates of mammalian target of ERK, JNK, and p38, MAPK families. And as ELK1 (Ser383) changed much more significantly and the phosphorylation of JNK also downregulated under RTEL1 knockdown. Thus, we attempted to determine whether RTEL1 facilitates phosphorylation of JNK signaling pathway to activate ELK1. We tested the phosphorylation status of JNK and ELK1 (Ser383) in U87 and SF295 cell by using western blot analysis. As shown in Fig. 4C, Knockdown of RTEL1 significantly decreased the expression of p-JNK and the downstream p-c-JUN, as well as the phosphorylated ELK1. However, ectopic expression of RTEL1 increased the activation of JNK pathway and the p-ELK1. These results indicated that JNK/ELK1 (Ser383) might be a potential target of RTEL1.

### RTEL1 knockdown results in accumulation of ROS and inactivation of JNK

**Fig. 5:**
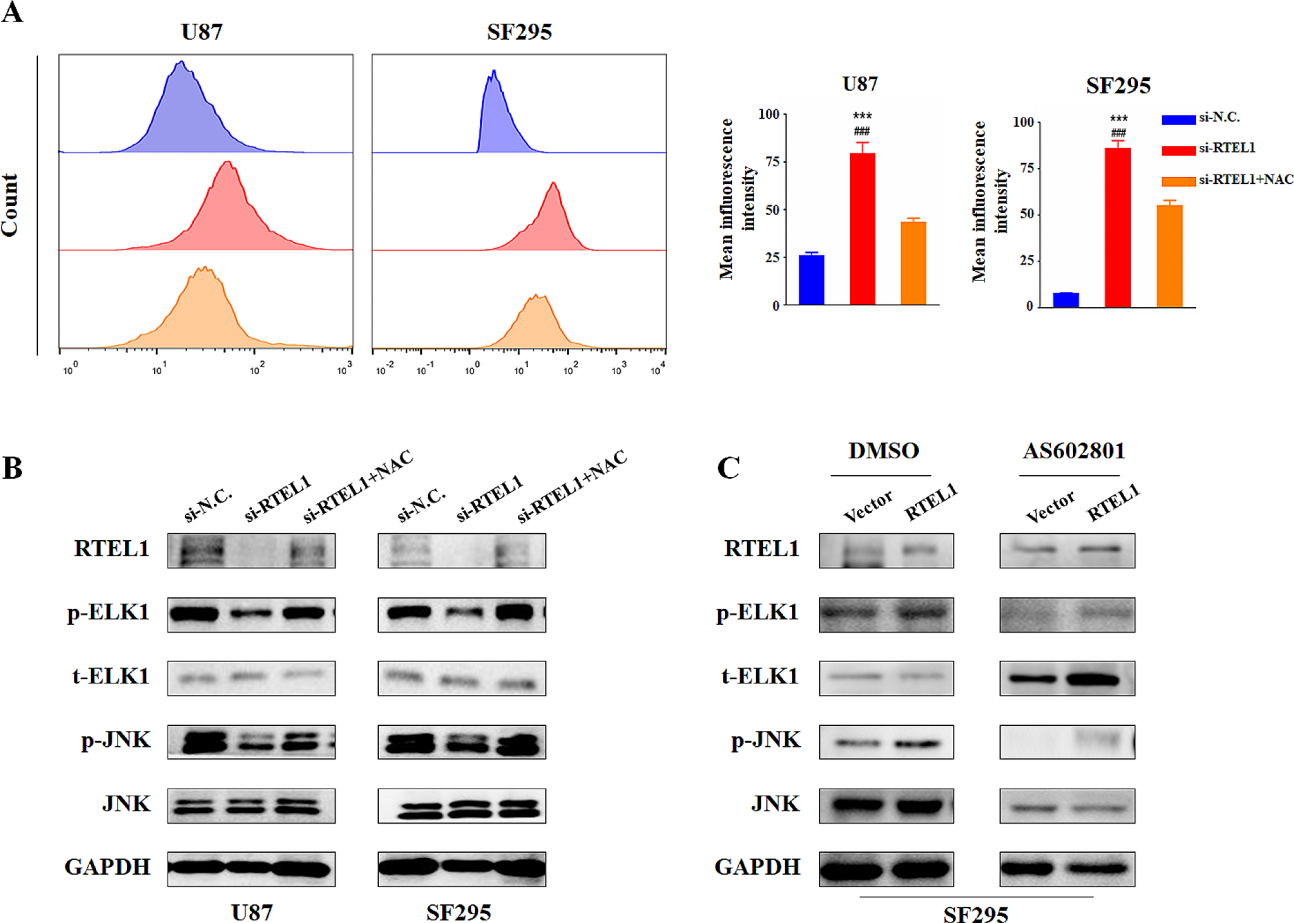
RTEL1 activated JNK signaling pathway and ELK1 in glioma cells. (**A**) ROS production was determined by flow cytometry. Knockdown of RTEL1 significantly promoted ROS release but abolished by ROS scavenger NAC. Mann–Whitney U test was used and data were expressed as mean ± SD. *** *p* < 0.001 represents si-N.C *v.s* si-RTEL1; ^###^*p* < 0.001 represents si- RTEL1 *v.s* si-RTEL1 + NAC. (**B**) Western blot analysis of RTEL1 Knockdown combination with NAC treatment in U87 and SF295 cells with GAPDH as the normalized controls. (**C**) Western blot analysis of RTEL1 overexpression combination with AS602801 treatment in SF295 cells with GAPDH as the normalized controls. si-N.C represents siRNA of non-target as controls; si-RTEL1-1724 represents siRNA of RTEL1; NAC represents ROS scavenger N-acetyl-L-cysteine; AS602801 represents JNK inhibitor (10 μM)

**Fig. 6:**
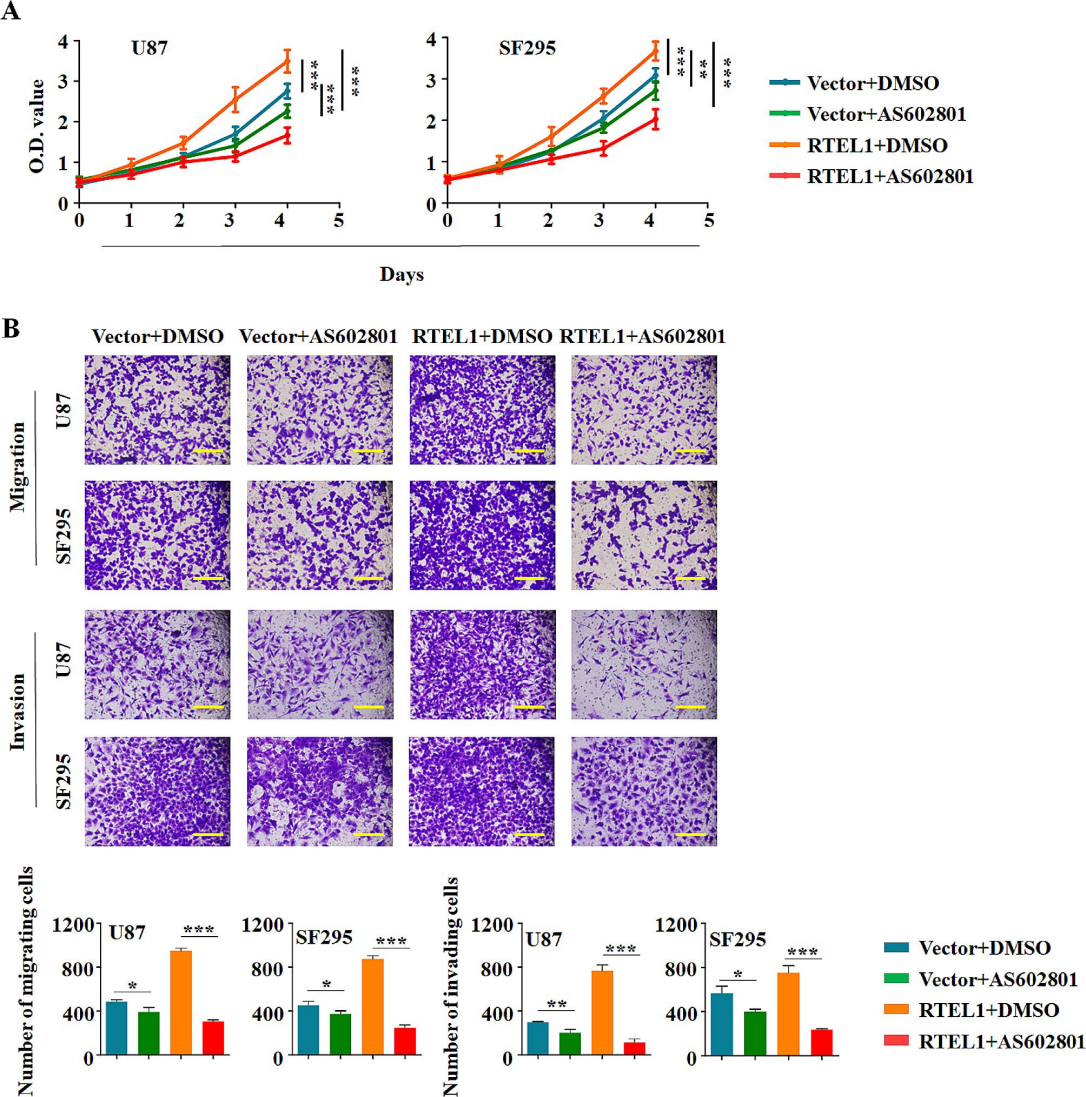
JNK inhibitor reverse the malignant biological of RTEL1 overexpression in glioma cells. (**A**) MTT assay was used to examine the proliferation effect of RTEL1 overexpression combination with AS602801 treatment in U87 and SF295 cells. JNK inhibitor AS602801 could significantly decreased cell proliferation ability up-regulated by RTEL1 overexpression both in U87 and SF295 cells. (**B**) The impact of RTEL1 overexpression combination with AS602801 treatment on colony formation ability of glioma cells U87 and SF295. JNK inhibitor AS602801 could significantly decreased cell migration and invasion ability up-regulated by RTEL1 overexpression both in U87 and SF295 cells. Vector represents empty plasmid pcDNA3.1(−); RTEL1 represents RTEL1 overexpression plasmid pcDNA3.1(−)-RTEL1; AS602801 represents JNK inhibitor (10 μM). Mann–Whitney U test was used and data were expressed as mean ± SD. * *p* < 0.05, ** *p* < 0.01, *** *p* < 0.001

Accumulating evidence showed that counterintuitively ROS can promote anti-tumourigenic signalling, initiating oxidative stress-induced tumour cell death. And recent review also showed a negative correlation between oxidative and telomere lengths. Thus, we attempted to determine whether ROS production was detected when RTEL1 knockdown in glioma cells. Our data showed that knockdown of RTEL1 significantly promoted ROS release, but this effect was abolished by treatment with a ROS scavenger, NAC (N-acetyl-L-cysteine; Fig. 5A). Accordingly, decreased phosphorylation of ELK1 and JNK caused by RTEL1 knockdown was rescued by NAC treatment in both U87 and SF 295 cells (Fig. 5B). The above data suggest that RTEL1 konckdown promotes glioma cell apoptosis through ROS-mediated cascade. What’s more, in order to determine whether JNK pathway also plays a key role in regulating ELK1 under RTEL1 overexpression in glioma cancer cells, JNK inhibitor AS602801 (10 μM) was used and the above phosphorylation effect was reversed in SF295 cells (Fig. 5C). Additionally, as shown in Fig. 6, we also found that JNK inhibitor AS602801 could significantly decreased cell proliferation, migration and invasion ability up-regulated by RTEL1 overexpression both in U87 and SF295 cells. Collectively, we demonstrated that RTEL1 could increase the phosphorylated ELK1 through activate the JNK signaling pathway in glioma cancer cells.

### RTEL1 knockdown inhibits glioma cell proliferation in vivo

**Fig. 7:**
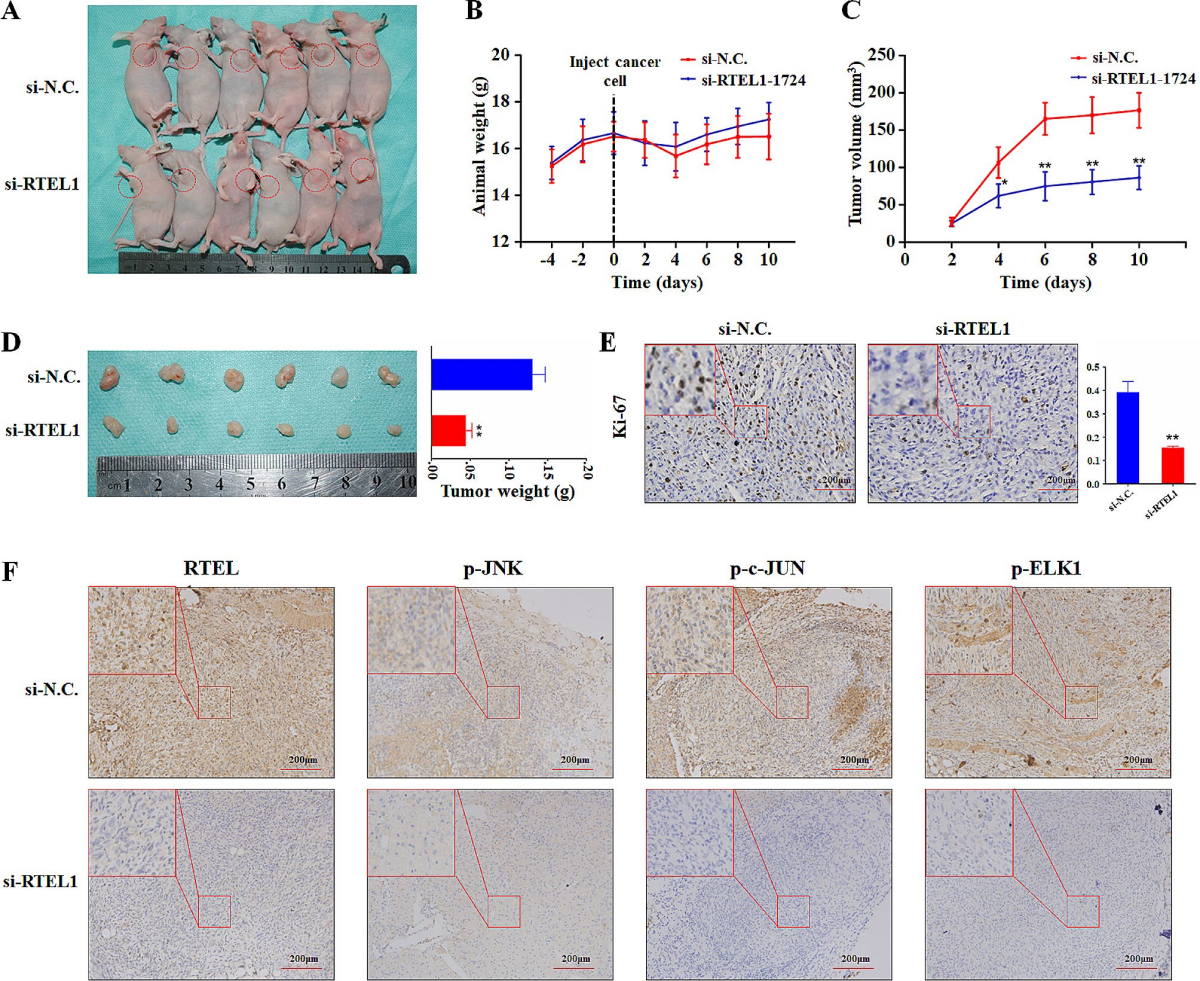
RTEL1 knockdown inhibits tumor growth in nude mice. Dissected tumors in vivo (**A**), growth curves (**B**), tumor volume (**C**) and mean tumor weight (**D**) of the indicated groups. Mann–Whitney U test was used and data were expressed as mean ± SD. * *p* < 0.05, *p* ** <0.01. (**E**) Representative Ki-67 staining of xenograft tumors from the indicated groups by immunohistochemistry staining images. (**F**) RTEL1 knockdown inhibits the proteins level of p-JNK signaling and p-ELK1 in vivo, detected by immunohistochemistry (IHC) staining images. si-N.C represents siRNA of non-target as controls; si-RTEL1 represents siRNA of RTEL1; Scale bar, 200 μm

Lastly, we also evaluated in vivo tumorigenic ability of RTEL1 in nude mice. RTEL1 knockdown significantly suppressed tumor growth compared with the control (Fig. 7A). Moreover, xenograft tumors were rapidly isolated and weighted at the end of the experiments. The average weight of RTEL1 knockdown group was significantly reduced compared to the control group (Fig. 7B). Meanwhile, we analyzed Ki-67 expression (a marker of proliferative cells) to determine the impact of RTEL1 on cell proliferation in vivo. As expected, the percentage of Ki-67-positive cells was significantly declined in the RTEL1 knockdown group compared to the control group (Fig. 7C). Altogether, our findings supported the strong tumorigenic role of RTEL1 in glioma. To further validate regulatory effect of RTEL1 on phosphorylation of JNK and ELK1 in vivo, we performed immunohistochemistry (IHC) assay using the sections of the above xenograft tumors. The results confirmed that RTEL1 knockdown significantly decreased phosphorylation of JNK, c-JUN and ELK1 compared to the tumors (Fig. 7D). Taken together, our results suggested that RTEL1 attenuates ROS accumulation, which downregulates JNK pathway and ELK1 phosphorylation, consequently modulate downstream targets of ELK1 in glioma cancer cells.

## Discussion

The most conclusive prognostic factors for glioma are extent of tumor resection, age at diagnosis, and Karnofsky performance status [1, 2, 29, 30]. Recent advances in molecular diagnostic techniques provide alternative methods for tumor classification using molecular abnormalities and signaling pathways involved in glioma genesis [1, 2, 29–32]. Point mutations in the TERT gene promoter, leading to increased telomerase activity, are found in 75% of oligodendrogliomas and primary glioblastomas [33–35]. Gliomas that do not carry TERT promoter mutations frequently harbor mutations of the telomere binding protein, activating the pathway of alternative lengthening of telomeres (ALT). A GWAS study identified two SNPs within intron 12 (rs6010620) and intron 17 (rs4809324) of RTEL1 that are significantly associated with glioma and astrocytoma predisposition [36]. Similarly, two further glioma GWAS studies, revealed a significant association with SNP (rs6010620) in intron 12 of the RTEL1 gene, and amplification of the 20q13.33 region was observed in nearly 30% of gliomas, with copy-number changes correlating with RTEL1 expression levels [37, 38]. Interestingly, Ubiquitous overexpression of RTEL1 in the mouse specifically caused hepatocellular tumors that recapitulated a variety of malignant features of human hepatocellular carcinoma (HCC) [25]. However, its role and exact mechanism in human cancers including glioma still has not been elucidated until now. In this study, we provided strong evidences supporting that RTEL1 is a potent oncogene in glioma both in vitro and in vivo. First, we demonstrated that RTEL1 was frequently up-regulated in gliomas compared to matched non-cancerous tissues, and found the association of increased expression of RTEL1 with poor patient outcomes. Second, knocking down of RTEL1 in glioma cells showed significant growth-inhibitory effect by inhibition of cell proliferation, colony formation, migration, invasion, and tumorigenic potential in nude mice, and induction of cell cycle arrest and apoptosis. Conversely, overexpression of RTEL1 depletion significantly hindered cell proliferation and colony formation, further supporting its tumor genesis function.

To better understand tumor genesis activity of RTEL1, we took an RNA-sequence assay, the result showed that RTEL1 knockdown resulted in upregulation or downregulation of numerous target genes. The genes are associated with 3 categories, including “transmembrane signal receptor activity”, “plasma lipoprotein particle”, and “lectin pathway”. The top 20 KEGG enrichment pathways contain olfactory transduction, cytokine-cytokine receptor interaction, JAK-STAT signaling pathway, neuroactive ligand-receptor interaction, cell adhesion molecules, drug metabolism-cytochrome P450. What’s more, to verify the downstream target gene, we conducted phospho-specific antibody microarray. We identified ELK1(Ser383) might be a key downstream gene regulated by RTEL1 in U87 and SP295 cells. We then evaluated the effect of RTEL1 on aberrant signaling of the JNK pathways in glioma cells. Knockdown of RTEL1 significantly decreased phosphorylated JNK and ELK1. And ectopic expression of RTEL1 increased this pathway. By using JNK inhibitor AS602801, we found the above phosphorylation effect was reversed and AS602801 could also decreased cell proliferation, migration and invasion ability up-regulated by RTEL1 overexpression. Collectively, our results showed that RTEL1 significantly facilitated phosphorylation of JNK signaling and the downstream transcription factor ELK1 in the tumorigenesis of glioma cells. What’s more, increased reactive oxygen species (ROS) production has been detected in various cancers and has been shown to have several roles [39]. ROS can activate pro-tumourigenic signalling, enhance cell survival and proliferation, and drive DNA damage and genetic instability [40, 41]. However, counterintuitively ROS can also promote anti-tumourigenic signalling, tumour cells express elevated levels of antioxidant proteins to detoxify elevated ROS levels [42], establish a redox balance, while maintaining pro-tumourigenic signalling and resistance to apoptosis [43–46]. Our data showed that knockdown of RTEL1 significantly promoted ROS release, but this effect was abolished by treatment with NAC. Besides, decreased phosphorylation of JNK/ELK1 caused by RTEL1 knockdown could rescued by NAC treatment. This data suggests that RTEL1 konckdown promotes glioma cell apoptosis through ROS-mediated cascade.

**Fig. 8:**
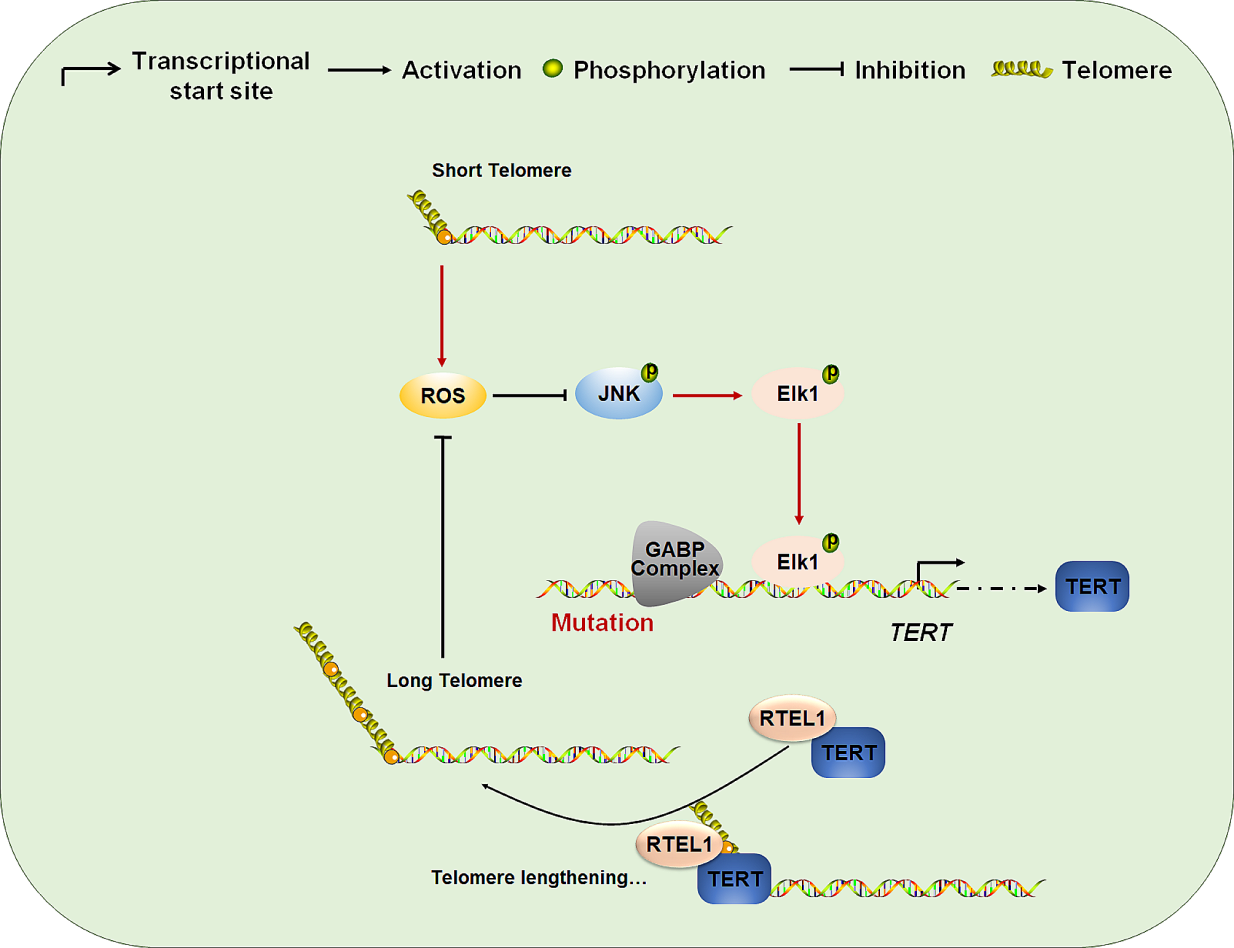
Schematic model of RTEL1/JNK/ELK-1 signaling-induced tumorigenesis of glioma. Accumulation of ROS caused by short telomere or RTEL1 Konckdown will suppress the JNK/ELK1 signaling pathway in glioma cells. RTEL1 cooperated with TERT maintains telomere elongation. The long telomere or ROS scavenger will decrease the ROS level and re-active the JNK caspase to promote TERT transcription. In conclusion, RTEL1 might promote glioma tumorigenesis through JNK/ELK1 cascade and ROS signaling

In conclusion, our study indicated that RTEL1 mRNA expression was positive correlation with telomere length and predicates worse progression in glioma patients. We also found that RTEL1 might promote glioma tumorigenesis through JNK/ELK1 cascade and ROS signaling (Fig. 8).

### Electronic supplementary material

Below is the link to the electronic supplementary material.

Supplementary Material 1

Supplementary Material 2

Supplementary Material 3

Supplementary Material 4

## Data Availability

No datasets were generated or analysed during the current study.
