# Supplementary material for: High expression of RTEL1 predicates worse progression in gliomas and promotes tumorigenesis through JNK/ELK1 cascade
Source: BMC Cancer. 2024 Mar 26;24:385. doi: 10.1186/s12885-024-12134-8 (PMC10967111; doi:10.1186/s12885-024-12134-8)
Supplement: Supplementary file 1 — Supplementary Material 1 [file 12885_2024_12134_MOESM1_ESM.docx]

Supplementary Table 1. The sequences of oligonucleotides of siRNAs

| si-RNAs | Sequences (5′-3′) |
| --- | --- |
| si-NC (Sense) | UUCUCCGAACGUCUCACGUTT |
| si-NC (Antisense) | ACGUGACACGUUCGGAGAATT |
| si-NCFAM (Sense) | UUCUCCGACACGUUCACGUTT |
| si-NC FAM (Antisense) | ACGUGACACGUUCGGAGAATT |
| si-RTEL1-1724 (Sense) | GGCGGACAUUAUCCAGAUUTT |
| si-RTEL1-1724 (Antisense) | AAUCUGGAUAAUGUCCGCCTT |
| si-RTEL1-1250 (Sense) | CCGCAGAGCACACAACAUUTT |
| si-RTEL1-1250 (Antisense) | AAUGUUGUGUGCUCUGCGGTT |
| si-RTEL1-3794 (Sense) | GCUGACAGCCUAGAAGCAATT |
| si-RTEL1-3794 (Antisense) | UUGCUUAUAGGCUGUCAGCTT |

Supplementary Table 2. RNA-sequence assay results of the differential expression genes

Supplementary Table 3. Phospho-specific microarray results of the differential changed genes

Supplementary Table 4. The primers sequence for RT-qPCR

| Gene | Sequences（5′-3′） |
| --- | --- |
| tel（Sense） | GGTTTTTGAGGGTGAGGGTGAGGGTGAGGGTGAGGGT |
| tel（Antisense） | TCCCGACTATCCCTATCCCTATCCCTATCCCTATCCCTA |
| HBG （Sense） | GCTTCTGACACAACTGTGTTCACTAGC |
| HBG（Antisense） | CACCAACTTCATCCACGTTCACC |

Supplementary Table 5. Association of the expression of RTEL1 and the RTL with clinicopathological characteristics in gliomas (n =389)

| Characteristics | RTL | | |  | *RTEL1* | | |
| --- | --- | --- | --- | --- | --- | --- | --- |
|  | ≤0.62(%) | >0.62(%) | *P* |  | ≤3.98(%) | >3.98(%) | *P* |
| No. of Patients | 143 (36.8) | 246 (63.2) |  |  | 110 (28.8) | 279 (71.2) |  |
| Gender |  |  |  |  |  |  |  |
| Male | 84 (38.9) | 132 (61.1) | 0.343 |  | 49 (22.7) | 167 (77.3) | 0.007 |
| Female | 59 (34.1) | 114 (65.9) |  |  | 61(35.3) | 112 (64.7) |  |
| Age, years |  |  |  |  |  |  |  |
| Mean | 45 | 45 | 0.833 |  | 49 | 43 | 0.086 |
| SD | 13.1 | 14.8 |  |  | 14.2 | 13.5 |  |
| WHO grade |  |  |  |  |  |  |  |
| I | 11 (33.3) | 22 (66.7) | 0.685 |  | 5 (15.2) | 28 (84.8) | 0.061 |
| II | 65 (40.1) | 97 (59.9) |  |  | 39 (24.1) | 123 (75.9) |  |
| III | 45 (35.4) | 82 (64.6) |  |  | 43 (33.9) | 84 (66.1) |  |
| IV | 22 (32.8) | 45 (67.2) |  |  | 23 (34.3) | 44 (65.7) |  |
| Localization |  |  |  |  |  |  |  |
| Frontal lobe | 59 (34.5) | 112 (65.5) | 0.083 |  | 51 (29.8) | 120(70.2) | 0.036 |
| Temporal lobe | 50 (35.5) | 91 (64.5) |  |  | 31 (22.0) | 110 (78.0) |  |
| Parietal lobe | 22 (32.8) | 45 (67.2) |  |  | 30 (44.8) | 37 (55.2) |  |
| Occipital lobe | 7 (30.4) | 16 (69.6) |  |  | 5 (21.7) | 18 (78.3) |  |
| Cerebellum | 12 (70.6) | 5 (29.4) |  |  | 4 (23.5) | 13 (76.5) |  |
| Others | 16 (38.1) | 26 (61.9) |  |  | 12(28.6) | 30 (71.4) |  |
| Pathological diagnosis |  |  |  |  |  |  |  |
| DA | 91 (36.8) | 156 (63.2) | 0.496 |  | 54 (26.7) | 193 (73.3) | <0.001 |
| OL | 14 (31.8) | 30 (68.2) |  |  | 12 (22.7) | 32 (77.3) |  |
| OA | 21 (45.6) | 25 (54.3) |  |  | 20 (41.3) | 26 (58.7) |  |
| GBM | 17 (32.7) | 35 (67.3) |  |  | 24 (32.7) | 28(67.3) |  |
| Recurrence^▼^ |  |  |  |  |  |  |  |
| Yes | 57 (28.6) | 142 (71.4) | 0.005 |  | 49 (30.7) | 150 (69.3) | 0.207 |
| No | 58 (44.3) | 73 (55.7) |  |  | 41 (23.7) | 90 (76.3) |  |
| KPS^▼^ |  |  |  |  |  |  |  |
| ≥80 | 61 (39.6) | 93 (60.4) | 0.105 |  | 39 (23.3) | 115 (76.7) | 0.536 |
| <80 | 54 (30.7) | 122 (69.3) |  |  | 51 (33.1) | 125 (66.9) |  |
| Seizures^▼^ |  |  |  |  |  |  |  |
| Yes | 44 (31.7) | 95 (68.3) | 0.349 |  | 32 (30.2) | 107 (69.8) | 0.168 |
| No | 71 (37.2) | 120 (62.8) |  |  | 58 (26.2) | 133 (73.8) |  |

| Radiotherapy^▼^ |  |  |  |  |  |  |  |
| --- | --- | --- | --- | --- | --- | --- | --- |
| Yes | 64 (32.3) | 143 (67.7) |  |  | 42 (20.3) | 165 (79.7) |  |
| No | 51 (38.3) | 82 (61.7) |  |  | 48 (36.1) | 85 (63.9) |  |
| Chemotherapy^▼^ |  |  |  |  |  |  |  |
| Yes | 50 (36.5) | 87 (63.5) |  |  | 35 (27.0) | 102 (73.0) |  |
| No | 65 (33.7) | 128 (66.3) |  |  | 55(28.5) | 138 (71.5) |  |
| *TERT* mutations^▼^ |  |  |  |  |  |  |  |
| Yes | 48 (42.9) | 64 (57.1) | 0.073 |  | 39 (34.8) | 53 (65.2) | <0.001 |
| No | 77 (32.4) | 161 (67.6) |  |  | 51 (21.4) | 187 (78.6) |  |

**Abbreviations**: Diffuse astrocytoma (DA); Oligodendroglioma (OL); Oligoastrocytoma (OA); Glioblastoma (GBM); Karnofsky performance status (KPS); ^▼^Only 330 patients have complete survival information.

Supplementary Table 6. Multivariable analysis of characteristics of glioma patients divided according to the expression of RTEL1 and the RTL (n =115)

| Characteristics | RTL | | |  | RTEL1 | |
| --- | --- | --- | --- | --- | --- | --- |
|  | OR*（95% CI） | | P |  | OR*（95% CI） | P |
| Gender | 1.33 （0.83-2.14） | 0.243 | |  | 2.23 （1.77-3.24） | 0.012 |
| Age^1^ | 2.24 （1.09-4.60） | 0.029 | |  | 1.74 （1.00-2.55） | 0.059 |
| WHO grade^2^ | 0.95 （0.67-1.33） | 0.751 | |  | 2.11 （0.96-2.36） | 0.065 |
| Pathological^3^ diagnosis | 0.95 （0.74-1.22） | 0.680 | |  | 3.19 （1.15-5.22） | 0.002 |
| Recurrence | 1.70 （1.00-2.89） | 0.049 | |  | 1.75 （0.84-2.72） | 0.452 |
| Seizures | 1.23 （0.75-2.01） | 0.417 | |  | 1.32 （0.95-2.66） | 0.155 |
| KPS^4^ | 1.32 （0.79-2.20） | 0.289 | |  | 1.52 （0.95-2.12） | 0.089 |
| IDH1 mutation | 1.45 （0.86-2.45） | 0.168 | |  | 0.99 （0.45-1.36） | 0.895 |
| MGMT methylation | 0.99 （0.60-1.61） | 0.951 | |  | 1.84 （0.57-1.72） | 0.452 |

*****OR: odds ratio with 95% confidence interval; ^1^Age (per 10 years); ^2^WHO grade (I; II; III; IV); ^3^Pathological diagnosis (DA, OL, OA, GBM); ^4^KPS (≥80; <80)
