# Supplementary material for: High expression of RTEL1 predicates worse progression in gliomas and promotes tumorigenesis through JNK/ELK1 cascade
Source: BMC Cancer. 2024 Mar 26;24:385. doi: 10.1186/s12885-024-12134-8 (PMC10967111; doi:10.1186/s12885-024-12134-8)
Supplement: Supplementary file 4 — Supplementary Material 4 [file 12885_2024_12134_MOESM4_ESM.pptx]

## Slide 1
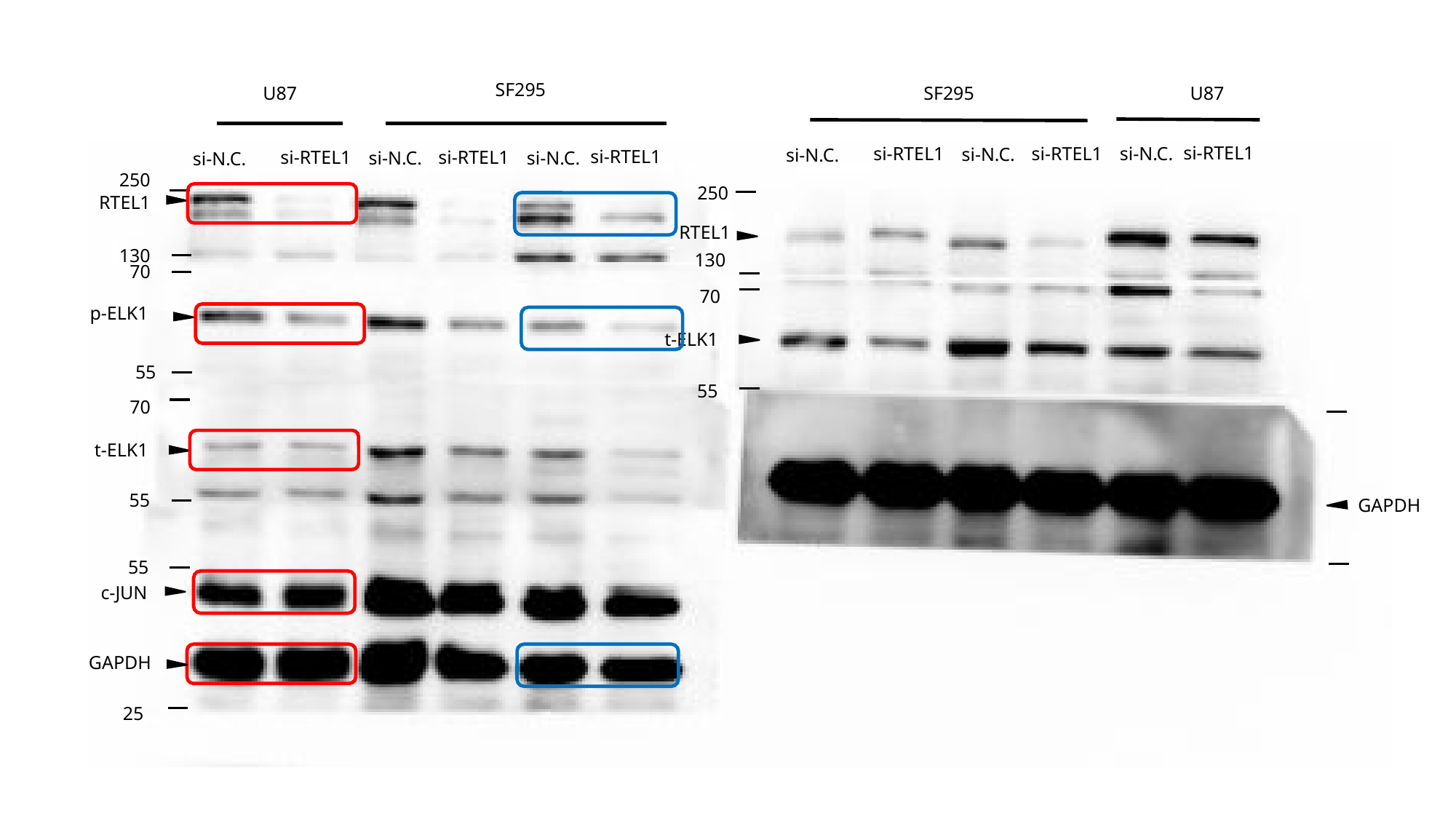

SF295
U87
U87
SF295
si-RTEL1
si-RTEL1
si-RTEL1
si-N.C.
si-N.C.
si-N.C.
si-RTEL1
si-RTEL1
si-RTEL1
si-N.C.
si-N.C.
si-N.C.
250
250
RTEL1
RTEL1
130
130
70
70
p-ELK1
t-ELK1
55
55
70
t-ELK1
55
GAPDH
55
25
55
c-JUN
GAPDH
25

## Slide 2
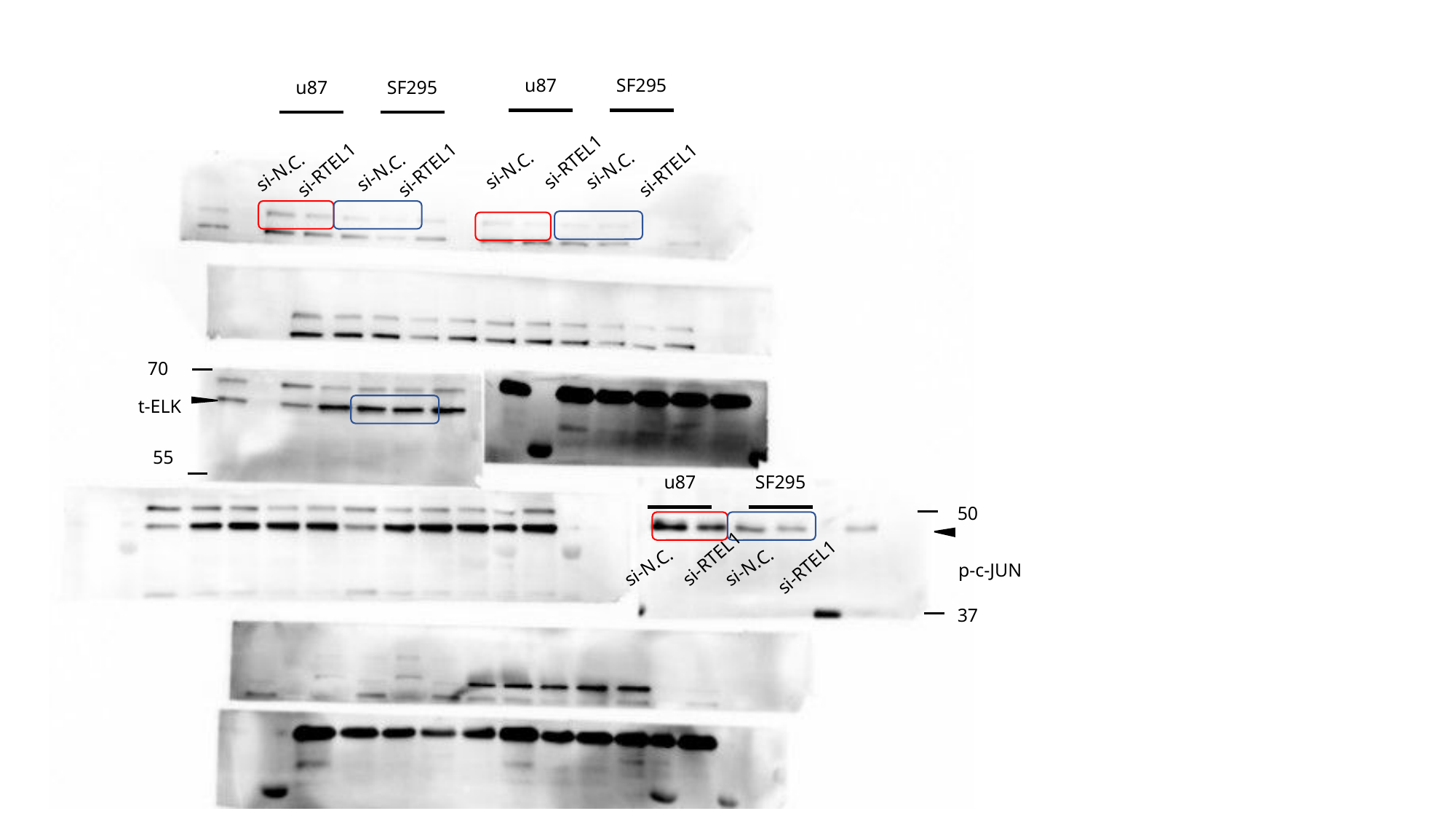

u87
SF295
u87
SF295
si-RTEL1
si-RTEL1
si-RTEL1
si-N.C.
si-N.C.
si-RTEL1
si-N.C.
si-N.C.
70
t-ELK
55
u87
SF295
50
si-RTEL1
si-N.C.
si-N.C.
si-RTEL1
p-c-JUN
37

## Slide 3
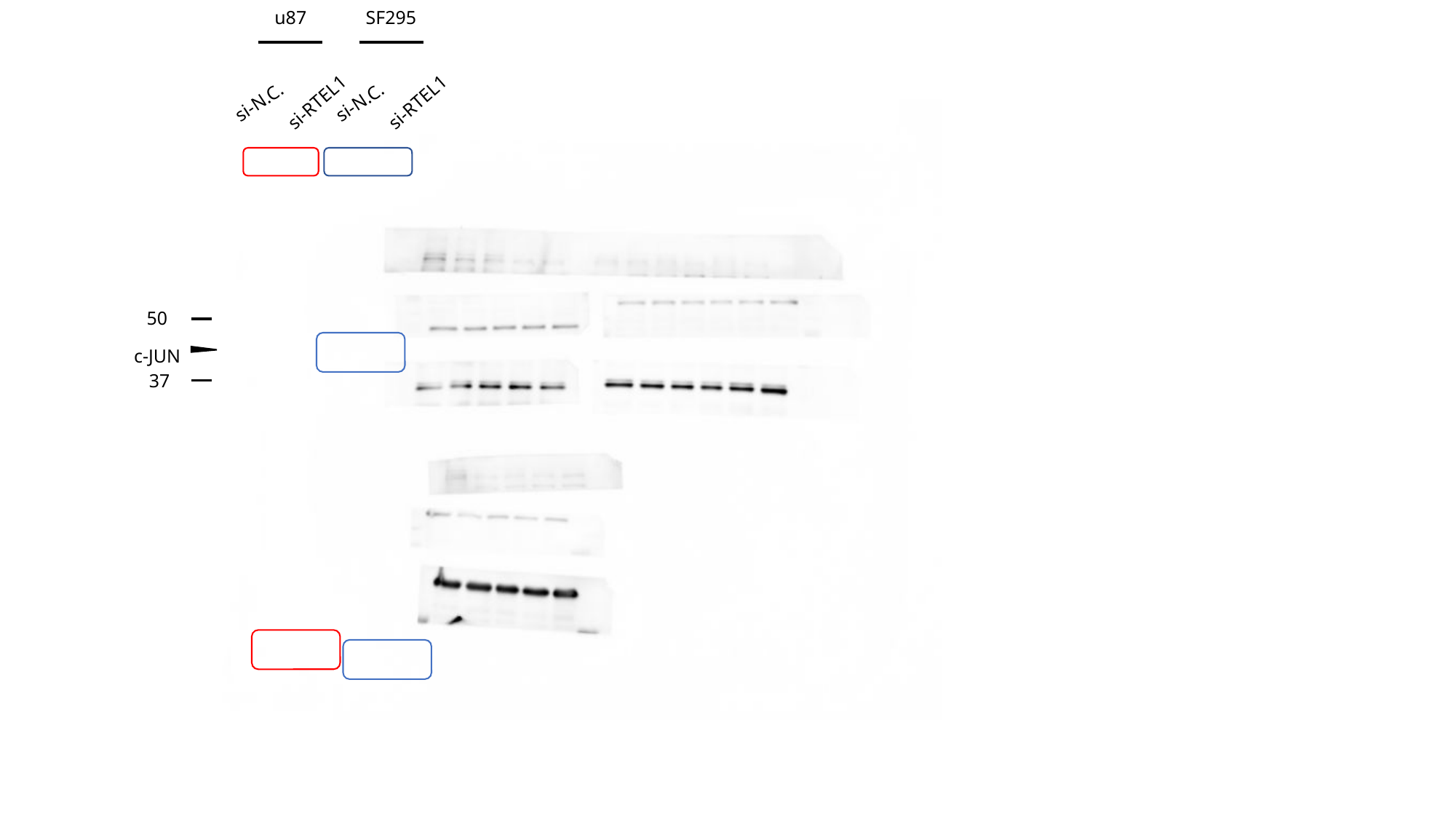

u87
SF295
si-N.C.
si-RTEL1
si-N.C.
si-RTEL1
50
c-JUN
37

## Slide 4
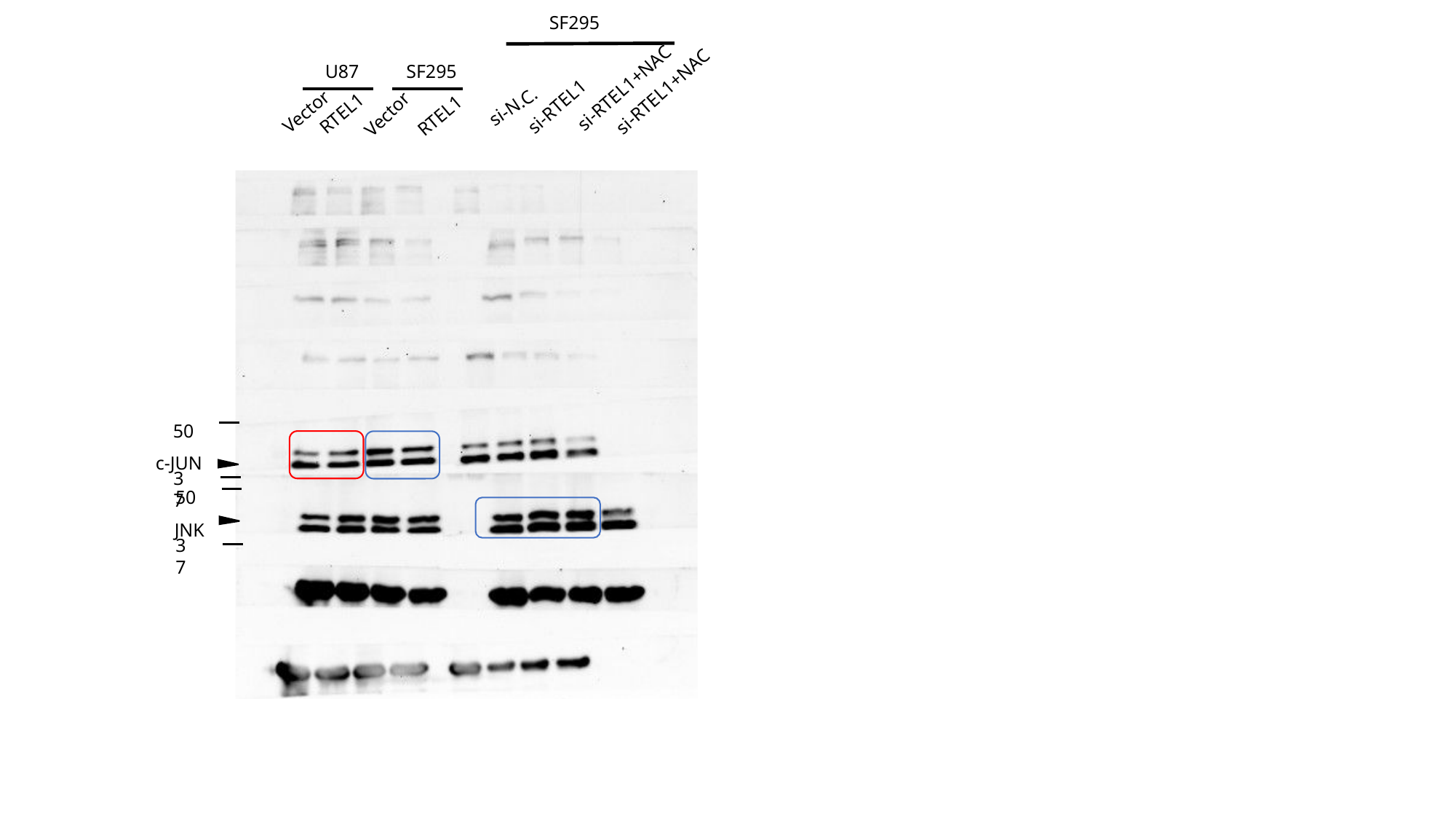

SF295
U87
SF295
si-RTEL1+NAC
RTEL1
si-RTEL1+NAC
Vector
si-N.C.
si-RTEL1
Vector
RTEL1
50
c-JUN
37
50
JNK
37

## Slide 5
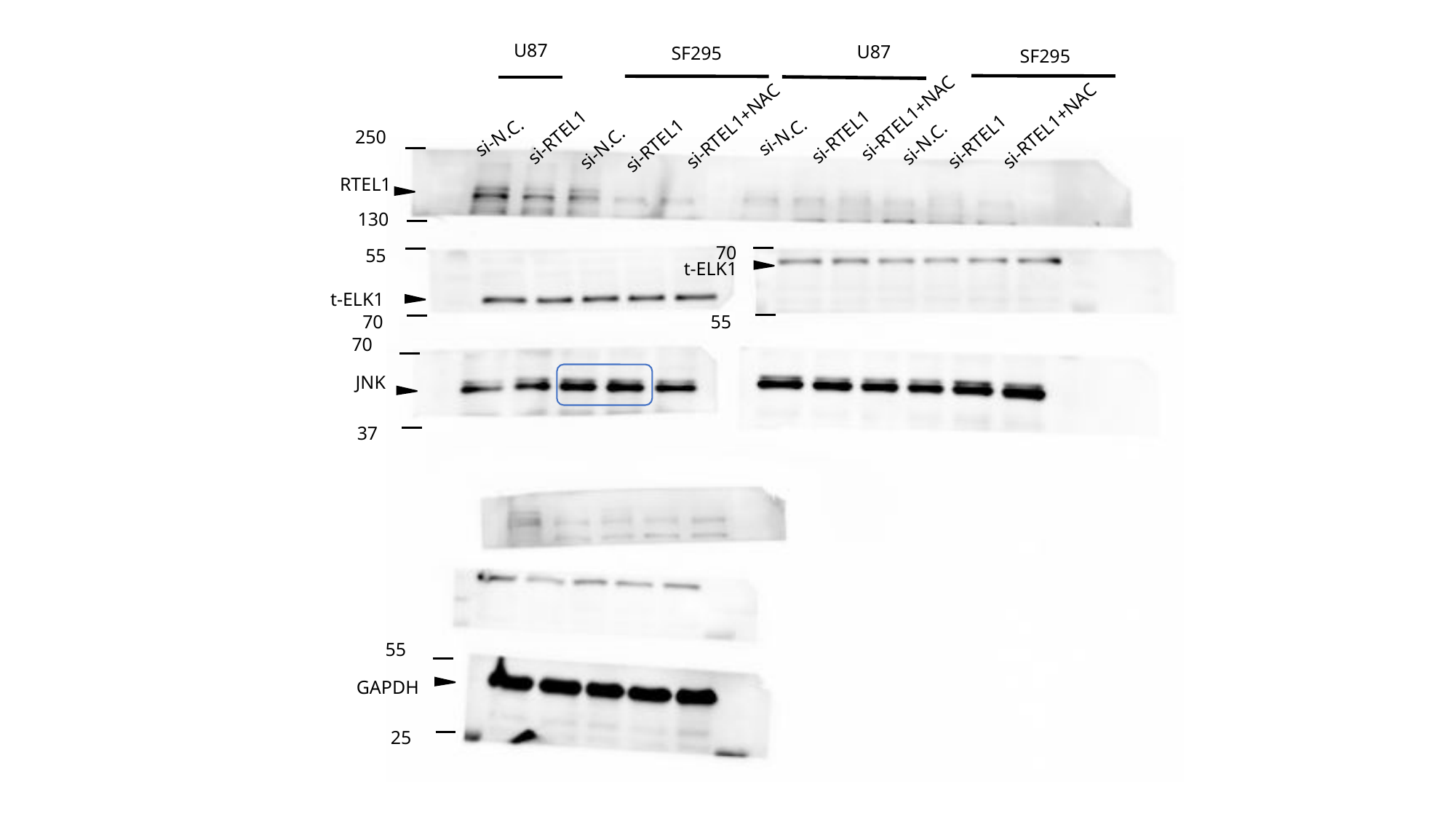

U87
U87
SF295
SF295
si-RTEL1+NAC
si-RTEL1+NAC
si-RTEL1+NAC
si-N.C.
si-RTEL1
250
si-N.C.
si-RTEL1
si-RTEL1
si-N.C.
si-RTEL1
si-N.C.
RTEL1
130
70
55
t-ELK1
t-ELK1
55
70
70
JNK
37
55
GAPDH
25

## Slide 6
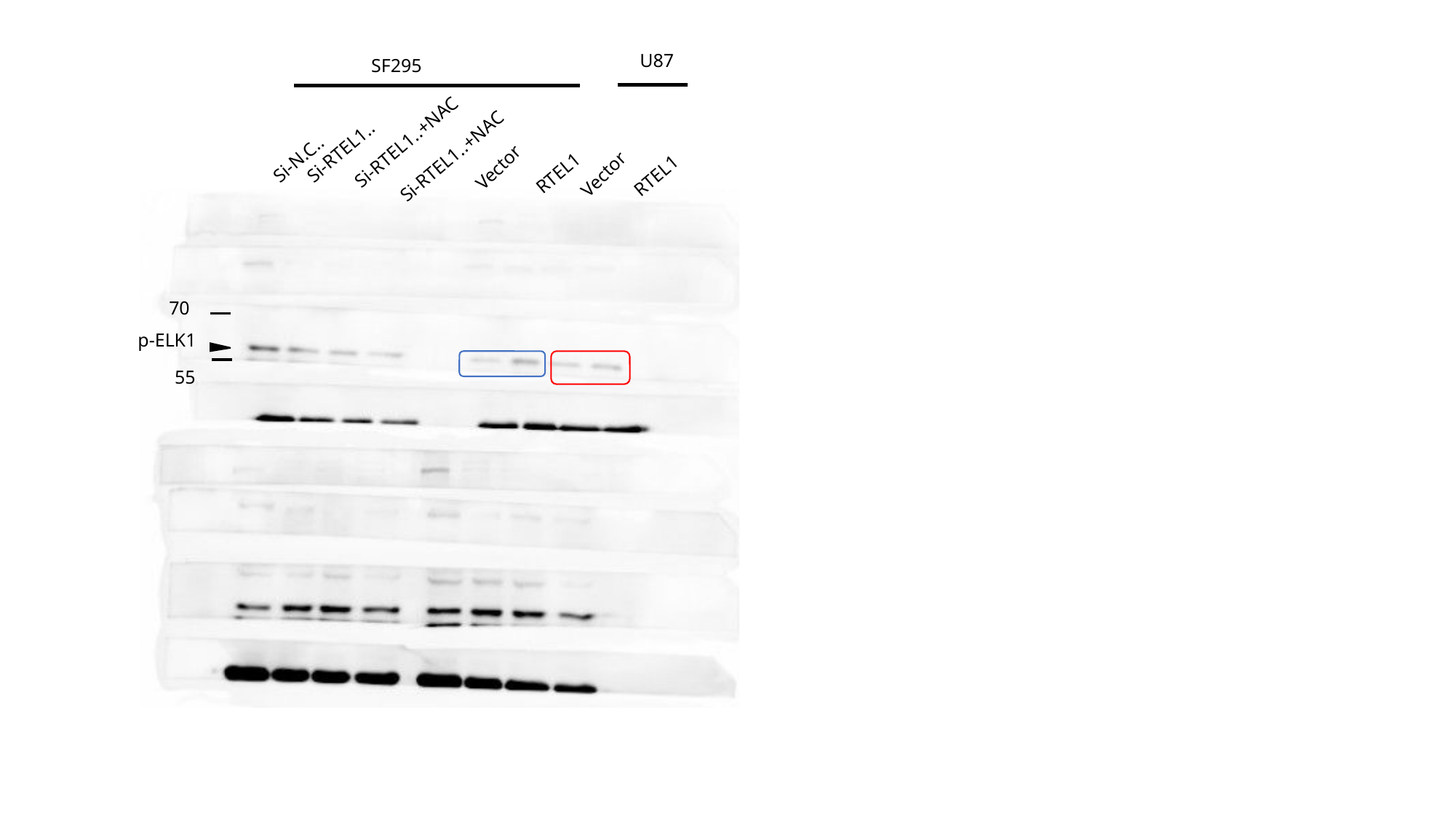

U87
SF295
Si-RTEL1..+NAC
Si-RTEL1..
Si-RTEL1..+NAC
RTEL1
Vector
Si-N.C..
Vector
RTEL1
70
p-ELK1
55

## Slide 7
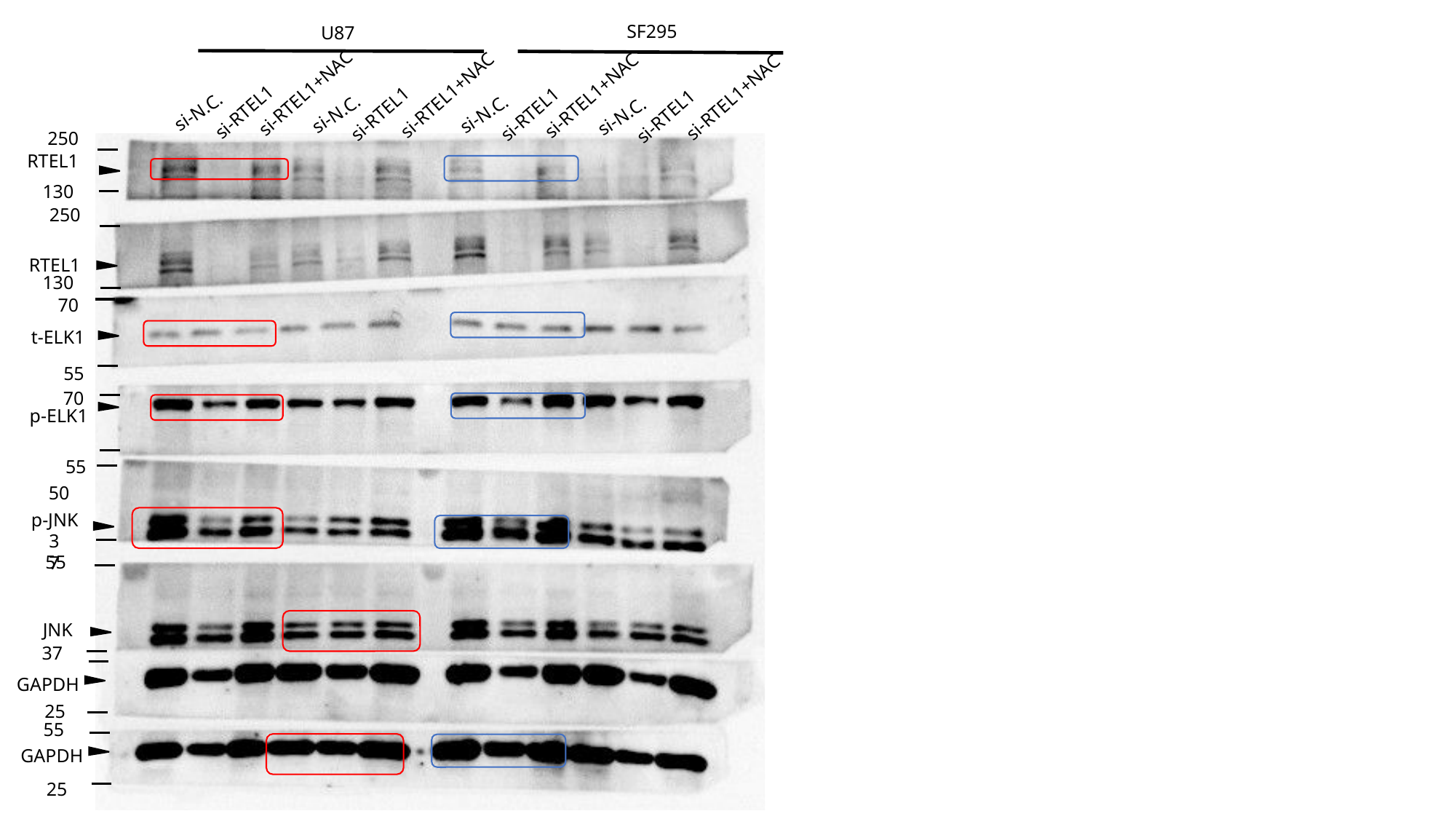

SF295
U87
si-RTEL1+NAC
si-RTEL1+NAC
si-RTEL1+NAC
si-RTEL1+NAC
si-N.C.
si-RTEL1
si-N.C.
si-RTEL1
si-N.C.
si-RTEL1
si-N.C.
si-RTEL1
250
RTEL1
130
250
RTEL1
130
70
t-ELK1
55
70
p-ELK1
55
50
p-JNK
37
55
JNK
37
GAPDH
25
55
GAPDH
25

## Slide 8
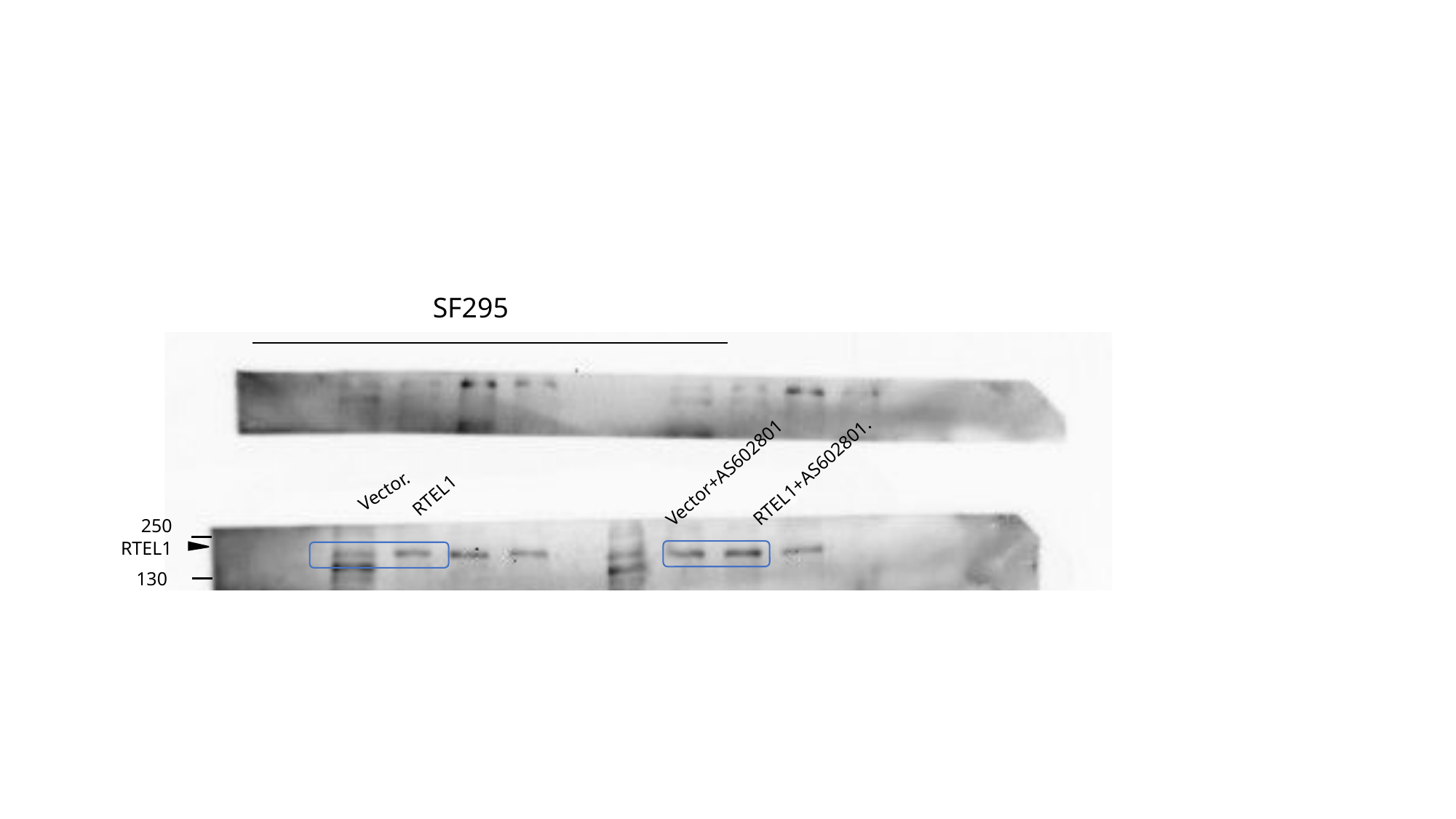

SF295
RTEL1+AS602801.
Vector+AS602801
Vector.
RTEL1
250
RTEL1
130

## Slide 9
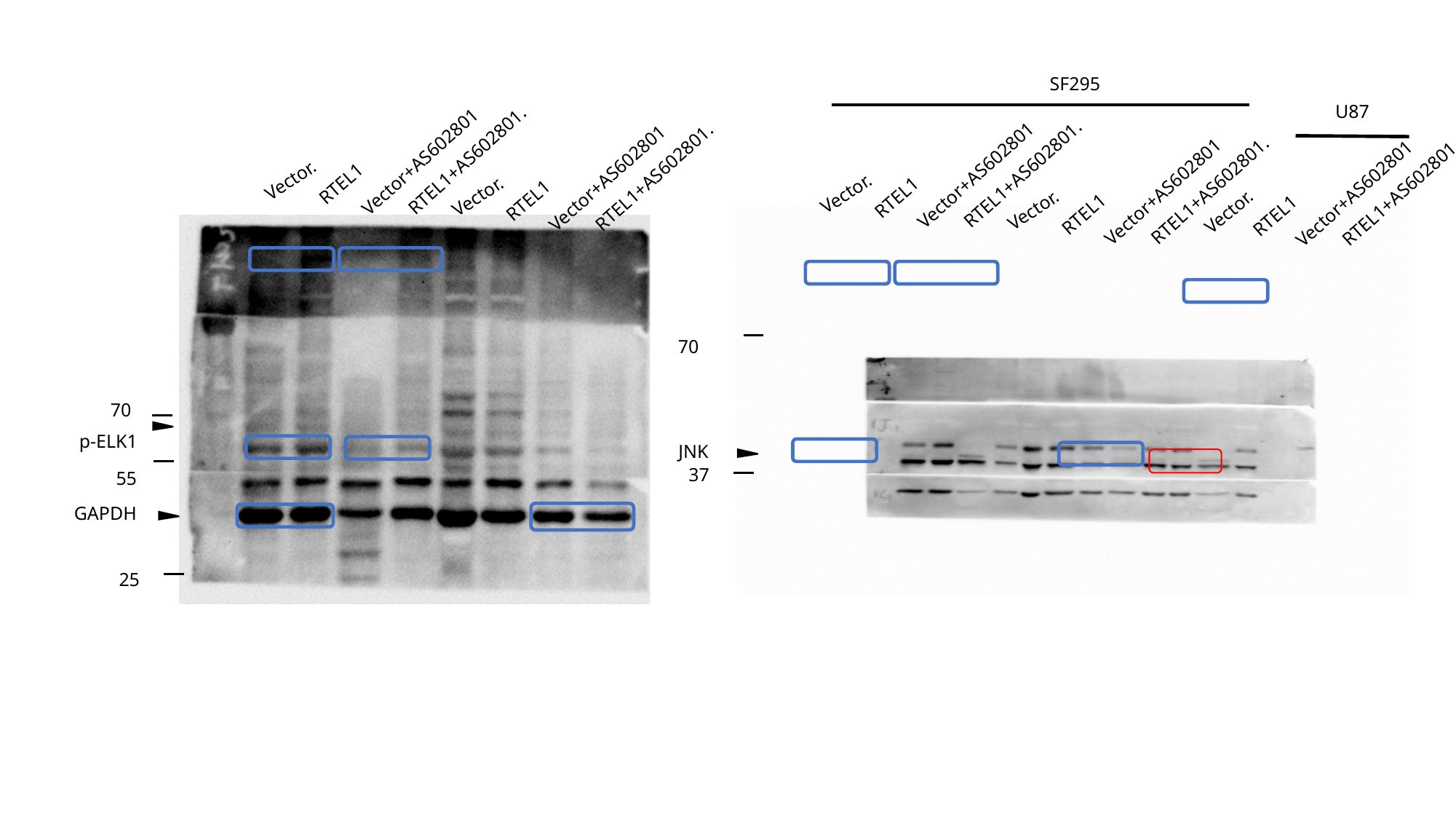

SF295
U87
RTEL1+AS602801.
Vector+AS602801
RTEL1+AS602801.
Vector+AS602801
RTEL1+AS602801.
Vector+AS602801
Vector.
RTEL1
Vector.
Vector.
RTEL1
RTEL1
70
JNK
37
RTEL1+AS602801.
Vector+AS602801
RTEL1+AS602801.
Vector+AS602801
Vector.
RTEL1
Vector.
RTEL1
70
p-ELK1
55
GAPDH
25

## Slide 10
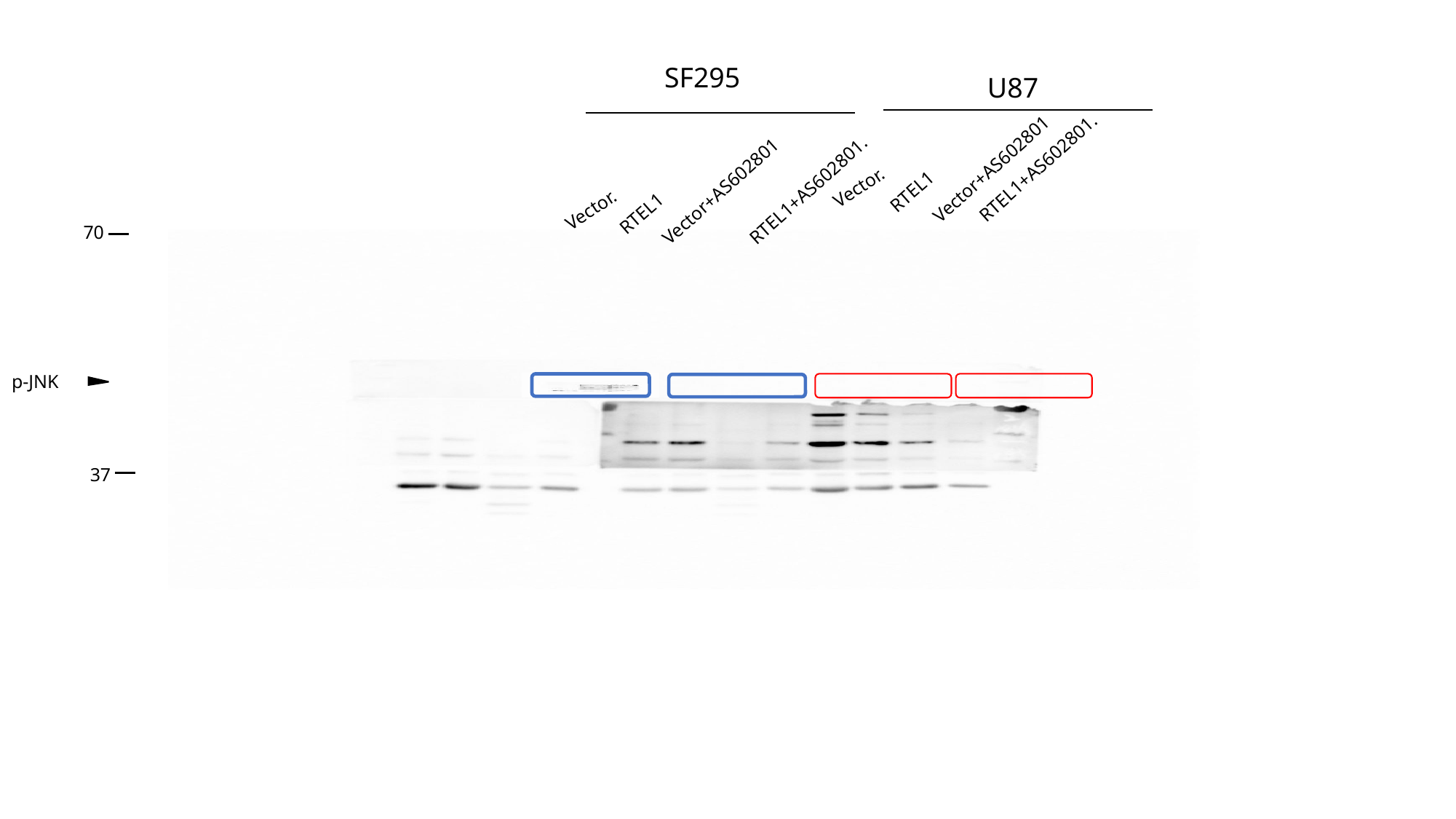

SF295
U87
RTEL1+AS602801.
Vector+AS602801
Vector.
RTEL1+AS602801.
Vector+AS602801
RTEL1
Vector.
RTEL1
70
p-JNK
37

## Slide 11
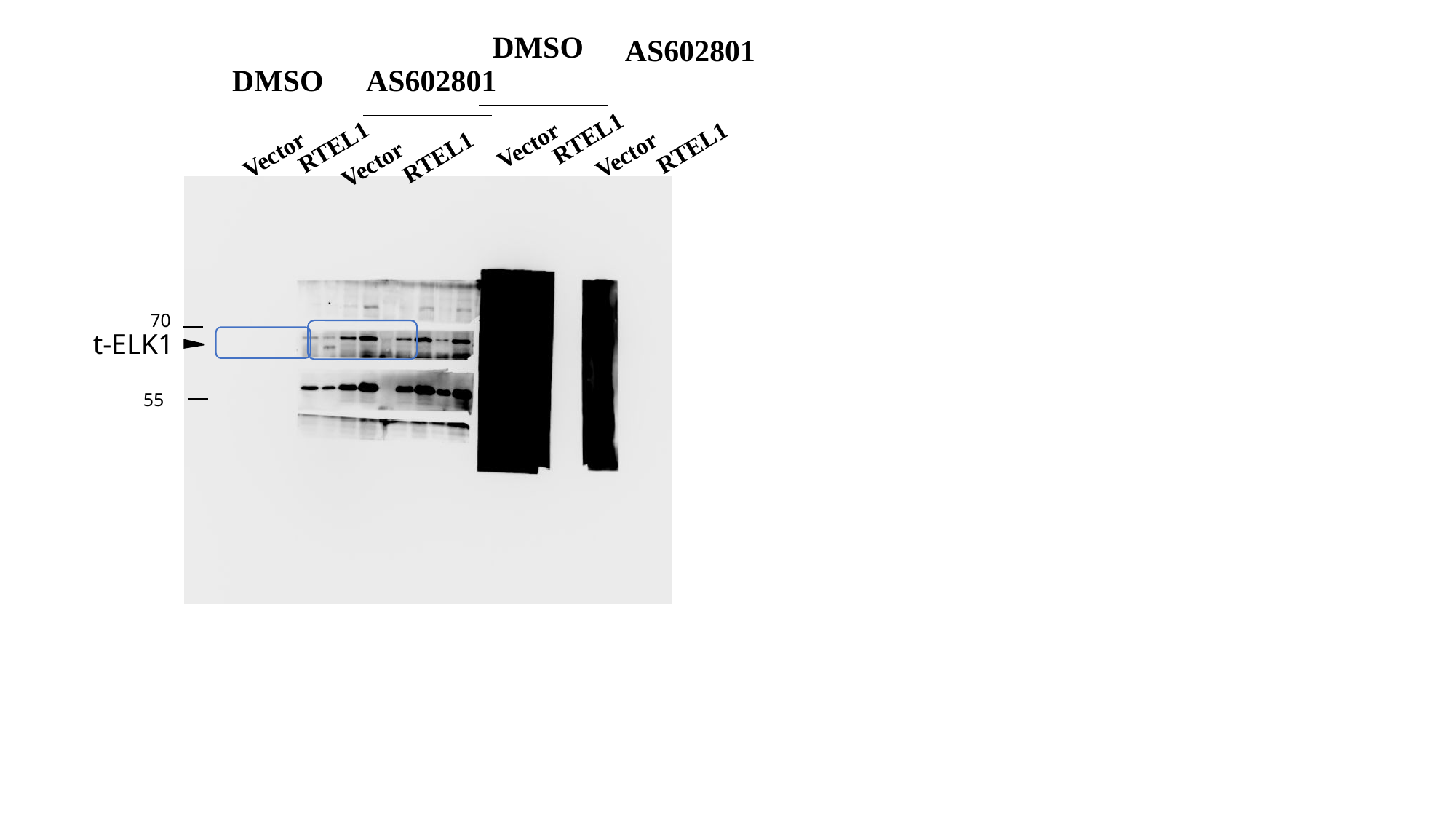

DMSO
AS602801
DMSO
AS602801
RTEL1
Vector
RTEL1
RTEL1
Vector
Vector
RTEL1
Vector
70
t-ELK1
55

## Slide 12
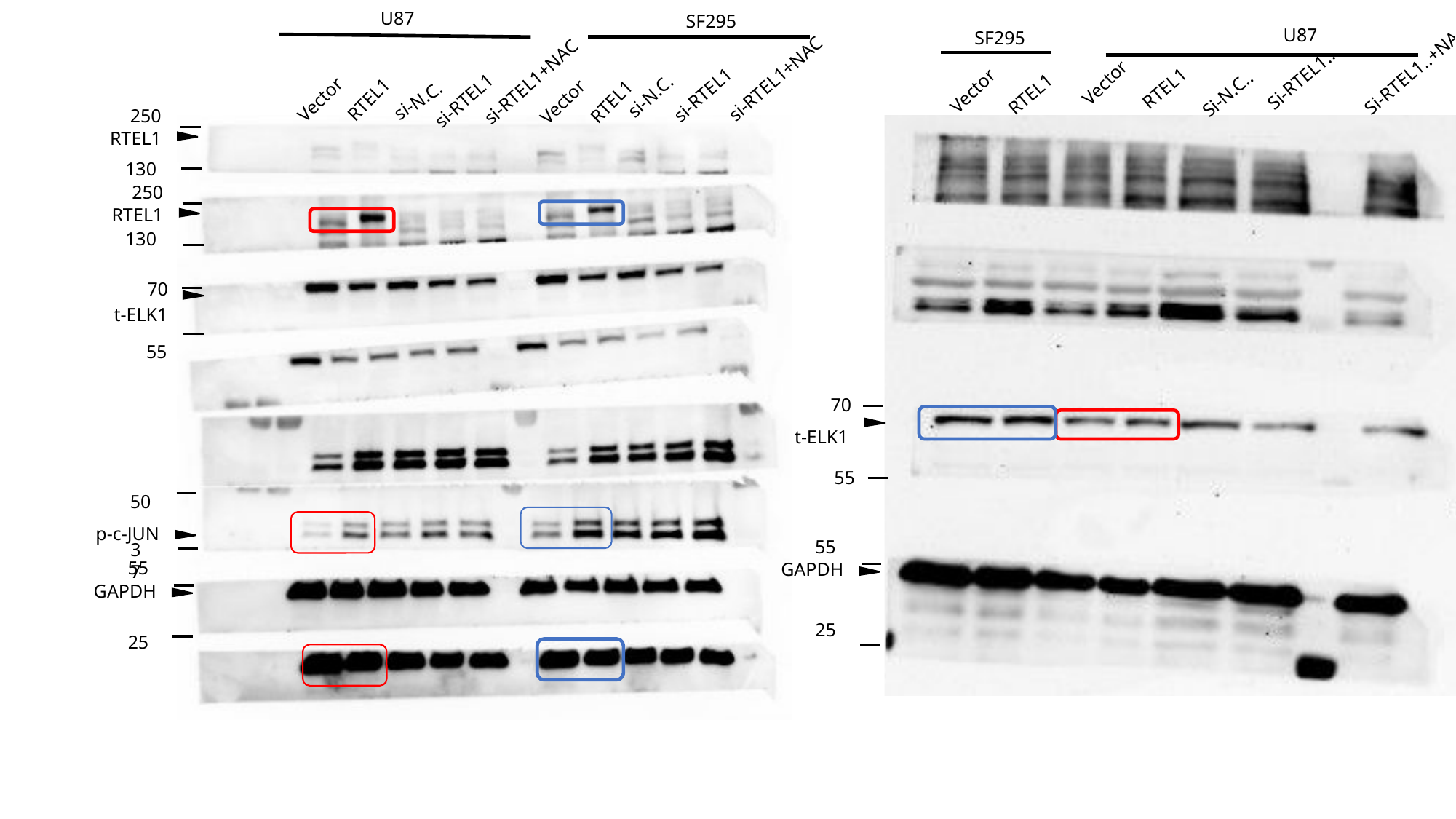

U87
SF295
U87
SF295
Si-RTEL1..+NAC
RTEL1
Vector
Si-RTEL1..
si-RTEL1+NAC
Vector
si-RTEL1+NAC
RTEL1
Si-N.C..
si-RTEL1
si-N.C.
RTEL1
Vector
si-N.C.
si-RTEL1
RTEL1
Vector
250
RTEL1
130
250
RTEL1
130
70
t-ELK1
55
70
t-ELK1
55
50
p-c-JUN
55
37
55
GAPDH
GAPDH
25
25

## Slide 13
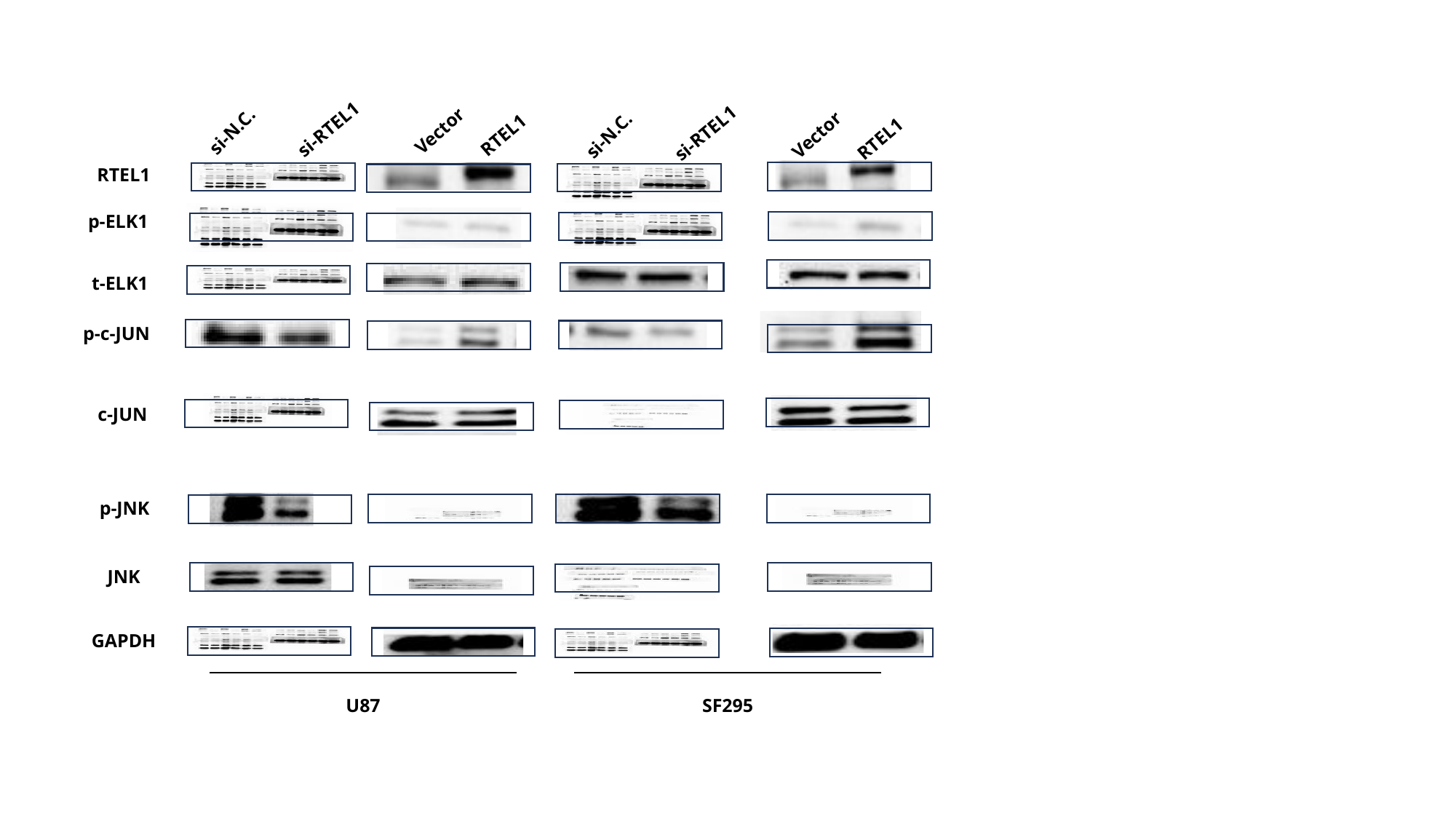

si-RTEL1
Vector
si-N.C.
si-RTEL1
Vector
RTEL1
si-N.C.
RTEL1
RTEL1
p-ELK1
t-ELK1
p-c-JUN
c-JUN
p-JNK
JNK
GAPDH
SF295
U87

## Slide 14
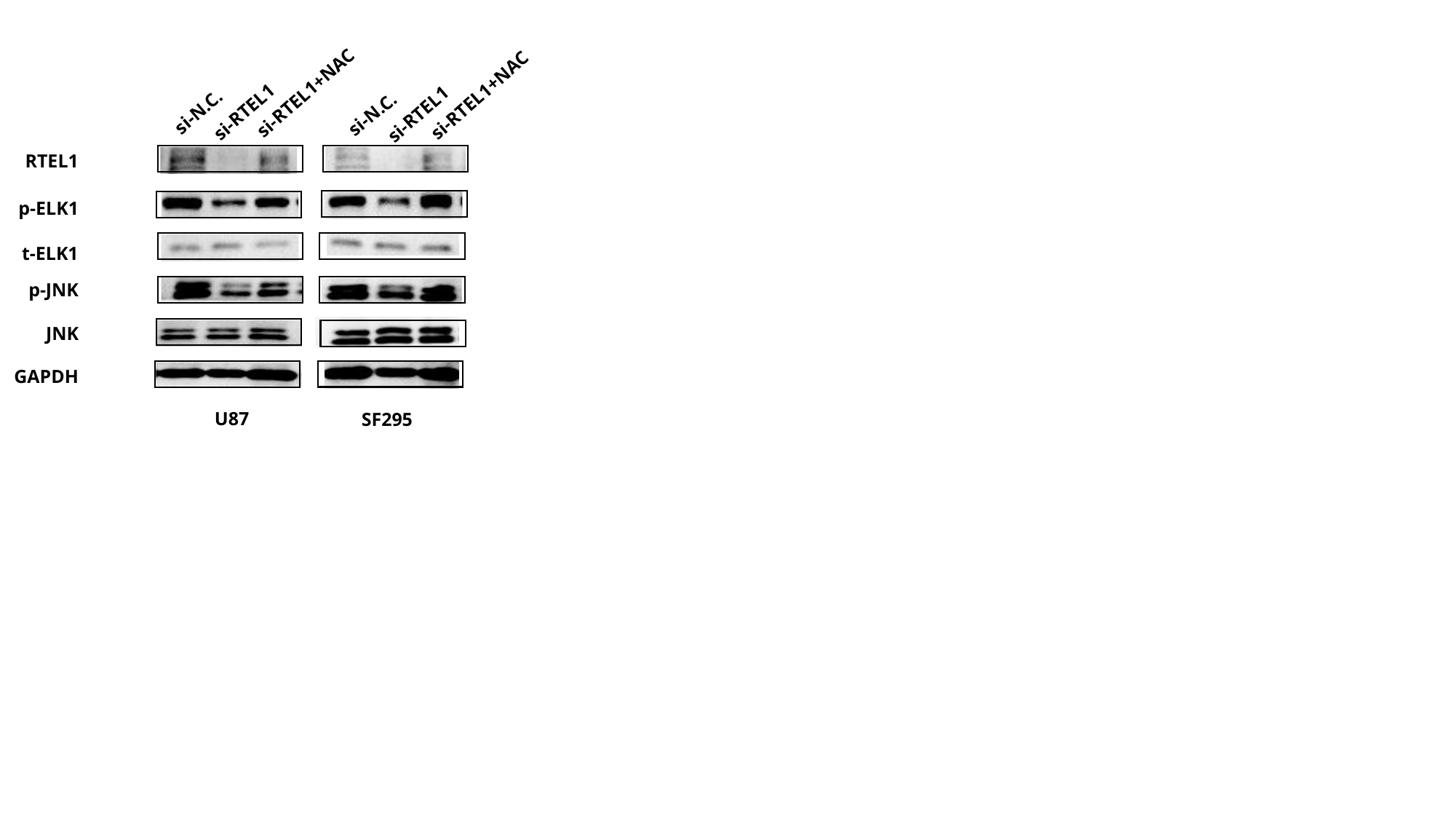

si-RTEL1+NAC
si-RTEL1+NAC
si-N.C.
si-RTEL1
si-N.C.
si-RTEL1
RTEL1
p-ELK1
t-ELK1
p-JNK
JNK
GAPDH
U87
SF295

## Slide 15
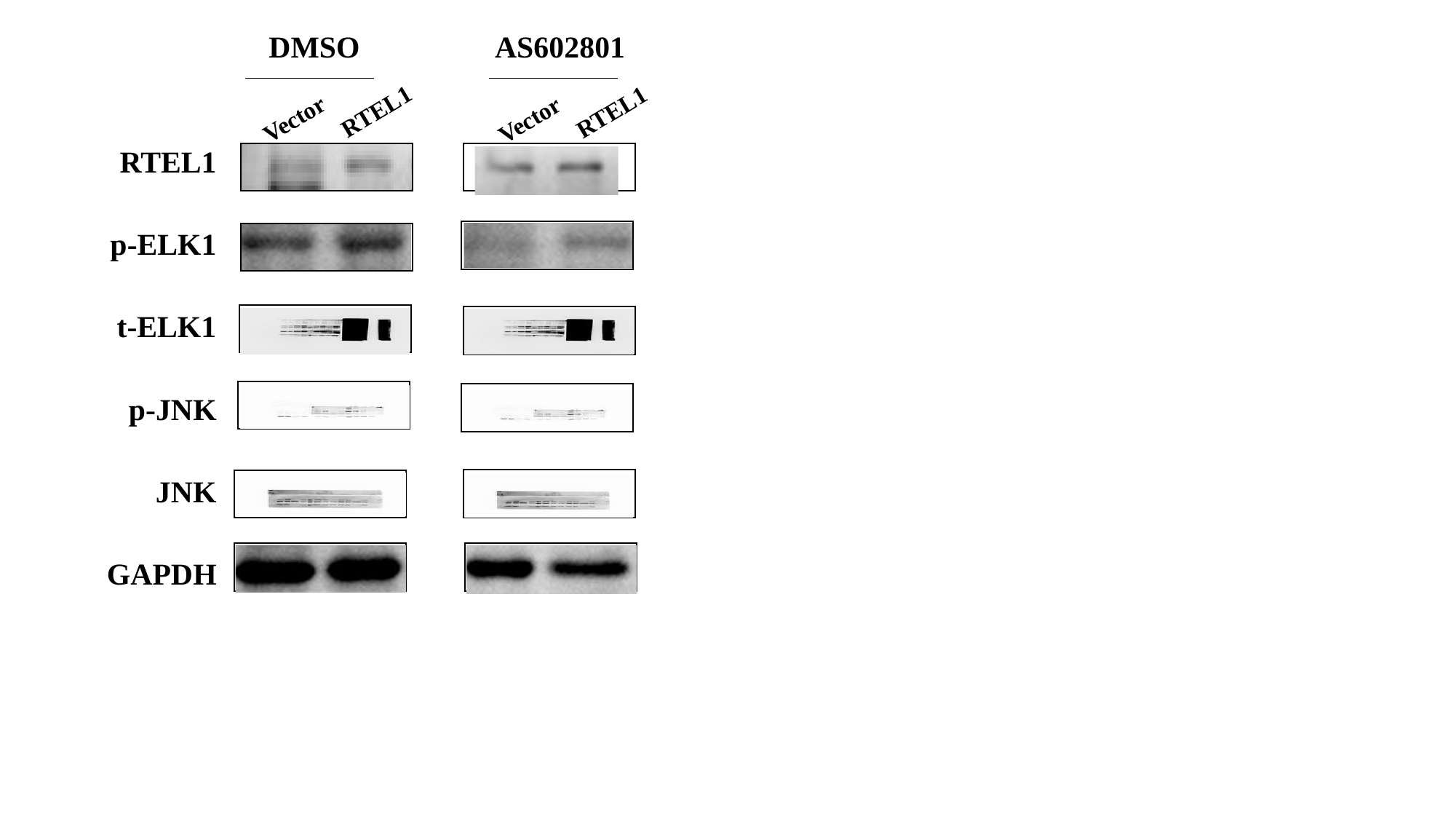

DMSO
AS602801
RTEL1
RTEL1
Vector
Vector
RTEL1
p-ELK1
t-ELK1
p-JNK
JNK
GAPDH
